# Supplementary material for: Development of an intervention to support reproductive health of garment factory workers in Cambodia: a qualitative study
Source: BMJ Open. 2021 Nov 22;11(11):e049254. doi: 10.1136/bmjopen-2021-049254 (PMC8611443; doi:10.1136/bmjopen-2021-049254)
Supplement: Supplementary data [file bmjopen-2021-049254supp001.pdf]

Appendix A: Interview guide for women seeking MA

Background information

What is your age?
Where are you from?
How long have you been in Phnom Penh?
Where do you work and what is your role? (Don't need specifics of factory)
How is your work?

Relationships and living situation

Are you married?
If no: do you have a relationship?
Do you have children?
How many?
How old are they?
Where do they live?
What sort of accommodation do you live in?
Who do you live with?

MA decision-making, expectations and information seeking

Have you had an abortion before?
When did you realise you were pregnant this time?
How did you realise you were pregnant?
What happened after that?<br>Feelings<br>Further confirmation sought<br>Who discussed it with<br>Why decided to have an abortion this time<br>Do you think this is a common reason for women to have abortion here?
How did you decide what to do?<br>Did you talk to your family or friends?

How did you make the decision?
Why did you choose this provider?
How did you learn about MA?<br>Do you have any friends or family who have had MA?<br>What was their experience?
Why did you choose MA instead of another way?

If have not taken MA yet:

Did you come with anyone today?
When do you think you will take the MA?
What do you expect to happen when you take the MA?
Have you been told will happen?<br>Who has told you this?<br>What has the provider said?
How will you manage it?
What will you do if you need more advice?
Will you need to take any time off work?
What are your feelings about taking it?

If have taken MA already:

Did you come with anyone to get the MA?
When did you take the MA?
What happened next?<br>If any decisions made or further care sought: ask about these and why
How did you manage the bleeding?<br>Did you use any products?<br>How much did these cost?
Did you take any time off work?
Was the MA process what you expected?<br>What did the provider tell you?

Did anyone else tell you what to expect?
What were your feelings about taking MA?
Did anyone help you at home?

Attitudes and barriers around MA

Was it easy or hard to come to buy MA?<br>Why was it easy/hard?<br>What would have made it better for you?
Who would you tell and who would you not tell?<br>Would you tell your friends?
Do people here think abortion is a bad thing?<br>Who says this?

If have not taken MA yet:

Do you know how much the cost is?<br>Will anyone help you pay?
Did you have to pay for transport here?

If have taken MA already:

What were the biggest challenges to taking MA?
How much did the MA (/MVA) cost?<br>Did anyone help you pay?<br>Did you have to pay for anything else? Transport?

Contraception

Have you used contraception before?
If yes: which methods?<br>How long for?<br>What was your experience of it?<br>Why did you stop using it?
If no: why do you not use contraception?

Do you plan to use any contraception after the abortion?
If yes: do you know which method you will have?<br>Do you know when you will get it?<br>Do you know where?<br>*If reported side effects:* do you know what you will do if you get the side effects?
If no: why not?<br>How will you prevent pregnancy?<br>Do you know which methods are available?
How do you make decisions about FP?<br>Where would you get information about FP?<br>Have you ever seen any videos or information online about FP?<br>Do you and your friends ever talk about contraception?<br>What contraception do your friends use?<br>What do they say about it?

General life questions

What are your plans for the future with your family and your work?
Do you have a mobile phone?<br>What type is it?<br>What do you use it for?
How do you enjoy yourself on your day off?

Is there anything you want to ask us?

Smith C, *et al. BMJ Open* 2022; 11:e049254. doi: 10.1136/bmjopen-2021-049254

Appendix B: Interview guide for private providers

MA provision

How long they have been providing MA
Available types of drug for MA
Approximate number of clients per week
Information given to clients

Training

What formal training they have been given
How they keep up to date
What support they would like

Post-abortion care and support

Post-abortion issues encountered
Post-abortion care provided
Suggestions for interventions

Smith C, *et al. BMJ Open* 2022; 11:e049254. doi: 10.1136/bmjopen-2021-049254
