# Supplementary material for: Development of an intervention to support reproductive health of garment factory workers in Cambodia: a qualitative study
Source: BMJ Open. 2021 Nov 22;11(11):e049254. doi: 10.1136/bmjopen-2021-049254 (PMC8611443; doi:10.1136/bmjopen-2021-049254)
Supplement: Supplementary data [file bmjopen-2021-049254supp002.pdf]

Supplementary file 2: video scripts

LOVE VIDEO: https://youtu.be/nVOSnsLvrBY	
**Character**	**Script**
BOPHA:	She is not married yet! Hmm... I would be afraid of getting pregnant!
SAMBO:	If I was her, I will use Condom in this situation.
MARDI:	But if you forgot to take the condoms. For me I would use injection every 3 months, so I do not worry about pregnancy anymore! Nobody knows I'm using it. It's easy to enjoy the romance.
LINDA:	I feel more confident by using the pill. My mother used it before she had me. It did not affect her health at all…
BOPHA:	... at least she had a very beautiful daughter!
HEANG:	I chose implant! Here it is! Implants are highly effective up to 3-5 years and discreet. I was not afraid to choose it as my older sister used it before. I will ask a provider to remove it only when I want to have a baby.
BOPHA:	Sorry girls, I have never used anything you talk about. I worry that in a spontaneous situation, I don't have any way to avoid unintended prenancy. Using contraception for protection is better than abortion.
Kunthea:	Hope is not a safe plan.This is my first child. I don't want to have another one right now. We need sometime to save money. You know, why I use IUD. IUD provides protection for up to 10 years long.It is small flexible plastic devide inserted into uterus. and it does not release Hormom like other method and i do not worry about pregnant when my husband back home i still have romance love as a couple on the rice field.
LADY IN RICEFIELD:	What method you choose is your choice. Keep calm and chose the contraceptive methods that fit the most with your life. Do not worry about getting pregnant!
VOICE OVER:	For more information about finding the right method that fits with for your health, or if contraception goes wrong and you need emergency contraception, call Marie Stopes to speak to an adviser<br>012 999 002/098 999 102 . Or find us on FB at https://www.facebook.com/MarieStopesKh

BABY DANCING VIDEO: https://youtu.be/RhmXnc-lvNs	
**Character**	**Script**
Doctor:	If you have any issue, come and talk to us in order to find a solution
Lady:	I feel hot and cold. Do not want to use pill. Even when I use it, don't use it regularly.
Doctor:	If you don't feel good, you should prevent in advance. Don't be careless to have unintended pregnancy. Prevention is better.
Lady:	It seems too early. It is weird for me without husband but using contraception
Doctor:	You have the choice, between injection, implant, IUD and condom. it's simple and easy.
Lady:	I am satisfied taking the pill but I have to know that I have to take it everyday. Thank you doctor for accurate information! Now I have no doubt, less anxiety to use contraception and there are many family planning methods for everybody. You have to think and choose correctly. Don't let yourself have sadness or bad problem because do not use any prevention.
Doctor + lady:	Nothing is easier than prevention. It make you have a happy family and a better future.
Voice over:	For more information about finding the right method that fits with for your health, or if contraception goes wrong and you need emergency contraception, call Marie Stopes to speak to an adviser 012 999 002/098 999102 . Or find us on FB at https://www.facebook.com/MarieStopesKh

MOTHER VIDEO: https://youtu.be/1GzGDI61Ssc	
**Character**	**Script**
YOUNG BROTHER:	What's wrong mum?
MUM:	I just feel dizziness, nausea and hot

Smith C, *et al. BMJ Open* 2022; 11:e049254. doi: 10.1136/bmjopen-2021-049254

YOUNG BROTHER:	Why? Are you sick Mum?
MUM:	I'm fine, don't worry, I am not sick at all, ...I just take medicine to prevent having another child
SISTER:	Mum wants to say that she took contraceptive' pills
SISTER:	You must take 1 pill every day regularly for it towork. It contains 2 hormones, similar to those that are already found in every woman's body.
YOUNG BROTHER:	Ah...I see. Do you have any side effects Mum?
MUM:	Yes I do son.
SISTER:	Don't worry Mum. The side-effects often go away after 2-3 months. Not everybody gets side effects like you. Do you know, it makes woman have regular periods, and could make you have nice skin and clear acne.
YOUNG BROTHER:	Are there any other methods that more effective and safe?
SISTER:	Well I see 3 more options. Isn't it Mum?
SISTER:	Injections are effective for 3 months. Nobody knows you are using it. You might have some irregular bleeding at first then you might have lighter bleeding. This happens because the uterus lining is not building up. It is normal to disapear over time. It does not affect your health.
SISTER:	If you want a long term family planning Implant is a good choice. It is effective for 3-5 years. It is a small soft plastic pipe. It takes the provider only 5 minutes to insert Implant to put it under arm skin or remove it. And when you want to have another child you just go to see provider to remove it. When it is take out you can have the baby again as normal.
SISTER:	The Intra Uterine Device or IUD is effective for 10 years. It is small and does not release hormones. It stops sperm from meeting the eggs to fertilise. No surgery is required to insert and remove IUD. It makes woman have regular periods.
MUM:	Thank you kids for all your advises!
BABY:	Don't worry Mum! Be confident with your choice for our future. We are here supporting you. Now you know there are other contraception methods for you to choose.
VOICE OVER:	For more information about finding the right method that fits with for your health, or if contraception goes wrong and you need emergency contraception, call Marie Stopes to speak to an adviser<br>012 999 002/098 999102 . Or find us on FB athttps://www.facebook.com/MarieStopesKh
