# Supplementary material for: Development of an intervention to support reproductive health of garment factory workers in Cambodia: a qualitative study
Source: BMJ Open. 2021 Nov 22;11(11):e049254. doi: 10.1136/bmjopen-2021-049254 (PMC8611443; doi:10.1136/bmjopen-2021-049254)
Supplement: Author's manuscript [file bmjopen-2021-049254.draft_revisions.pdf]

BMJ Open

BMJ Open is committed to open peer review. As part of this commitment we make the peer review history of every article we publish publicly available.

When an article is published we post the peer reviewers' comments and the authors' responses online. We also post the versions of the paper that were used during peer review. These are the versions that the peer review comments apply to.

The versions of the paper that follow are the versions that were submitted during the peer review process. They are not the versions of record or the final published versions. They should not be cited or distributed as the published version of this manuscript.

BMJ Open is an open access journal and the full, final, typeset and author-corrected version of record of the manuscript is available on our site with no access controls, subscription charges or pay-per-view fees (http://bmjopen.bmj.com).

If you have any questions on BMJ Open's open peer review process please email info.bmjopen@bmj.com

BMJ Open

Development of an intervention to support reproductive health of garment factory workers in Cambodia

Journal:	*BMJ Open*
Manuscript ID	bmjopen-2021-049254
Article Type:	Original research
Date Submitted by the Author:	19-Jan-2021
Complete List of Authors:	Smith, Chris; Nagasaki University, Tropical Medicine and Global Health; London School of Hygiene and Tropical Medicine, Clinical Research Department<br>Sokhey, Ly; Marie Stopes International Cambodia<br>Tijamo, Camille Florence; Marie Stopes International Cambodia<br>McLaren, Megan; Marie Stopes International<br>Watkins, Justin; School of Oriental and African Studies (SOAS)<br>Amra, Ou; Marie Stopes International Cambodia<br>Masuda, Chisato; Nagasaki University<br>Oreglia, Elisa; Kings College London
Keywords:	Reproductive medicine < GYNAECOLOGY, PUBLIC HEALTH, QUALITATIVE RESEARCH, REPRODUCTIVE MEDICINE

SCHOLARONE™
Manuscripts

I, the Submitting Author has the right to grant and does grant on behalf of all authors of the Work (as defined in the below author licence), an exclusive licence and/or a non-exclusive licence for contributions from authors who are: i) UK Crown employees; ii) where BMJ has agreed a CC-BY licence shall apply, and/or iii) in accordance with the terms applicable for US Federal Government officers or employees acting as part of their official duties; on a worldwide, perpetual, irrevocable, royalty-free basis to BMJ Publishing Group Ltd ("BMJ") its licensees and where the relevant Journal is co-owned by BMJ to the co-owners of the Journal, to publish the Work in this journal and any other BMJ products and to exploit all rights, as set out in our licence.

The Submitting Author accepts and understands that any supply made under these terms is made by BMJ to the Submitting Author unless you are acting as an employee on behalf of your employer or a postgraduate student of an affiliated institution which is paying any applicable article publishing charge ("APC") for Open Access articles. Where the Submitting Author wishes to make the Work available on an Open Access basis (and intends to pay the relevant APC), the terms of reuse of such Open Access shall be governed by a Creative Commons licence – details of these licences and which Creative Commons licence will apply to this Work are set out in our licence referred to above.

Other than as permitted in any relevant BMJ Author's Self Archiving Policies, I confirm this Work has not been accepted for publication elsewhere, is not being considered for publication elsewhere and does not duplicate material already published. I confirm all authors consent to publication of this Work and authorise the granting of this licence.

For peer review only

Title page

Development of an intervention to support reproductive health of garment factory workers in Cambodia

Authors

Chris Smith[1,2]

Ly Sokhey[3]

Camille Tijamo[3]

Megan McLaren[4]

Caroline Free[5]

Justin Watkins[6]

Ou Amra[3]

Chisato Masuda[1]

Elisa Oreglia[7]

Affiliations

[1] School of Tropical Medicine and Global Health, Nagasaki University, Sakamoto 1-12-4, Nagasaki 852-8523, Japan

[2] Clinical Research Department, London School of Hygiene and Tropical Medicine, Keppel Street, London WC1E7HT, United Kingdom

[3] Marie Stopes International Cambodia

[4] Marie Stopes International, London, UK

[5] Department of Population Health, London School of Hygiene and Tropical Medicine, Keppel Street, London WC1E7HT, United Kingdom

[6] School of Oriental and African Studies (SOAS)

[7] Kings College London

Corresponding author

Chris Smith

Email: christopher.smith@lshtm.ac.uk

Tel: +81(0)80-8710-5309

School of Tropical Medicine and Global Health, Nagasaki University, Sakamoto 1-12-4, Nagasaki 852-8523, Japan

Trial registration: N/A

For peer review only - http://bmjopen.bmj.com/site/about/guidelines.xhtml

For peer review only

Abstract (284 words)

Objectives: The aim of this study was to describe the development of an intervention to support the reproductive health of garment factory workers in Cambodia.

Design: Informed by intervention mapping and qualitative approaches, we conducted a needs assessment with general and participant observation and semi-structured interviews, followed by intervention development activities including specifying possible behaviour change, designing the intervention and producing and refining intervention content.

Setting: The research was conducted by a multidisciplinary team with backgrounds in public health, linguistics, digital cultures and service delivery in a suburb of Phnom Penh where many garment factories cluster.

Participants: Garment factory workers in Cambodia; typically young women aged under 30 years who have migrated from rural areas to Phnom Penh city.

Outcomes: Analysis of reproductive health issues facing garment factory workers and metrics of videos developed.

Results: Our research identified some challenges that Cambodian garment factory workers experience regarding contraception and abortion. Concerns or experience of side-effects were identified as an important determinant leading to non-use of effective contraception and subsequent unintended pregnancy. Financial constraints or a desire to space pregnancies were the main reported reasons to seek an abortion. Information about medical abortion given to women by private providers was often verbal, with packaging and the drug information leaflet withheld. Given the observed widespread use of social media among factory workers, we developed three short 'edutainment' videos about contraception which were evaluated after one month.

Conclusions: We describe the development of an intervention to support reproductive health among garment factory workers in Cambodia. These videos could be further improved and additional videos could be developed. More work is required to develop appropriate and effective interventions to support reproductive health of garment factory workers in Cambodia.

Keywords: Family planning, contraception, abortion, Cambodia, garment factory workers, intervention development, intervention mapping, videos, behaviour change

For peer review only

Strengths and limitations of the study

- This study sought to understand the reproductive health issues of a potentially vulnerable population of garment factory workers in Cambodia
- This study was conducted by a multidisciplinary team with backgrounds in public health, linguistics, digital cultures and service delivery
- We sought to employ participatory approaches in our needs assessment and intervention development
- Being a qualitative study, the number of participants was relatively small and may not be representative of garment factory workers or health providers
- It was challenging to explain and translate details of intervention mapping across languages, cultures and disciplines working in different paradigms

For peer review only

For peer review only - http://bmjopen.bmj.com/site/about/guidelines.xhtml

Development of an intervention to support reproductive health of garment factory workers in Cambodia

Background

Cambodia has made considerable improvements in economic and health indicators in the last two decades, with much of Cambodia's economic growth attributed to garment exports.(1)(2) In 2015, the estimated number of garment factory workers in Cambodia was estimated to be around 650,000, of whom 85% were female.(3)(4) Garment factory workers are potentially a vulnerable population in terms of support for reproductive health issues and access to services. A study of female factory workers conducted in 2014 reported that 80% were younger than 30 years, half had only primary school education, and most had migrated from rural areas away from their family and community support and did not have adequate access to affordable, high quality reproductive health information and services to meet their diverse needs.(5)

In Cambodia, reproductive health services are available through the public and private sectors, including non-governmental organisations (NGOs). A wide variety of contraception methods are available. Abortion was legalised in 1997 and medical abortion was approved by the Cambodian Ministry of Health in 2009.(6) Previous studies have examined medical abortion provision in clinics and pharmacies. A study in 2015 reported good knowledge of medical abortion among health care providers in clinics and pharmacies in Cambodia.(6) A study of clinical outcomes of medical abortion in 2019 reported no increased need for additional treatment among women seeking medical abortion from pharmacies compared with clinics, but that women were less likely to feel prepared for what happened after taking the pills or to be offered contraceptive methods when getting medical abortion pills. Users of the different services varied with 26% of women in the pharmacy group being garment factory workers compared with 9% in the clinic group.(7)

This current study was conceived based on the prior observation that many independent private healthcare providers are located in the vicinity of garment factories, but not much is known about what kind of reproductive health services they provide, particularly medical abortion and contraception, and what is the experience of clients who visit them. The project is a collaboration between Marie Stopes International Cambodia (MSIC) and three

For peer review only - http://bmjopen.bmj.com/site/about/guidelines.xhtml

universities in the United Kingdom. The study protocol and description of family planning practices are described elsewhere.(8)(9) The aim of this paper is to describe the development of an intervention to support the reproductive health of garment factory workers in Cambodia.

Methods

This research project was conducted during 2018-19 in a suburb of Phnom Penh where many garment factories cluster, with the co-operation of three factories and a number of private healthcare providers. We were a multidisciplinary team with backgrounds in public health, linguistics, digital cultures and service delivery. The research was conducted by different members of the research team at different times. Each member focused on their area of expertise and methods, while participating in data gathering done by other members. Findings from the different phases and approaches informed subsequent data gathering, in an iterative process aimed at understanding the wider context of medical abortion and contraception first (needs assessment), and develop possible interventions later. Research methods included general and participant observation, semi-structured interviews and periodic meetings in the UK and Cambodia to discuss findings.(8)

The methods were informed by intervention mapping methods,(10) but in a more holistic manner, which took into consideration the different disciplines involved. We aimed to conduct a needs assessment followed by intervention development activities including specifying possible behaviour change targets, designing the intervention and producing and refining intervention content. Coding of the data was done in both a deductive way, utilizing codes derived from the structure of the intervention mapping needs assessment process, and inductively, using codes that emerged from the more open-ended methods, including observations and semi-structured interviews with factory workers. The end result was not a pure application of intervention mapping but rather a modified approach to it.

In order to learn about the wider context of factory workers' choices regarding reproductive health, we conducted a short ethnographic study of their lives, living conditions, work places, and digital technology use. We joined several women during their lunch breaks in factory grounds, chatting about their experiences and getting a sense of who they were friends with, what they discussed during breaks, how they found their current jobs and what

For peer review only - http://bmjopen.bmj.com/site/about/guidelines.xhtml

their future plans were. We also shared meals with them in nearby restaurants after working hours and at weekends, and were able to visit some of their homes. After an initial period of "hanging out" we conducted semi-structured interviews that focused primarily on their mobile phone use, to understand what sites/apps they used, what content they consumed online and off, and whether they searched for specific information. Some of the interviews covered healthcare and health-related topics. We also did semi-structured interviews with owners of mobile phone shops and service providers near the factories, to triangulate what we learnt from the interviews about mobile phone use.

In order to learn about garment factory workers' use of reproductive health services we observed the practice of and interviewed garment factory infirmary workers and a variety of private healthcare providers ranging from simple drug stores to larger clinics. Interviews explored practices around reproductive health, especially with regards to contraception and abortion provision. With the co-operation of selected private providers we conducted interviews with women seeking abortion services. Interviews explored reasons for seeking abortion services and their experiences. Further details regarding recruitment, data collection and analysis are reported elsewhere.(9)

During the course of the project we organized several meetings to share and discuss behaviours observed in research that could feasibly be targeted and potential interventions resulting from the needs assessment activities, and then design, produce and refine a pilot intervention. In order to address limitations in the provision of accessible contraceptive information and information materials for factory workers in general and specifically at the time of seeking medical abortion we decided to make three 'edutainment' videos about family planning and one instructional video about how to take MA correctly. We collaborated with a Cambodian media company (Phare) and during the process sought input from garment factory workers in a pre-testing workshop.(11) The videos were designed to target barriers to contraception use that were identified in the needs assessment, ethnographic and interviews studies.(9) The videos were released through MSIC's communication channels and their impact was evaluated by an independent digital media company (Havas Champagne media).(12)

Ethics

For peer review only - http://bmjopen.bmj.com/site/about/guidelines.xhtml

Ethical approval was obtained from the Cambodia Human Research Ethics committee (number: 094NECHR), the London School of Hygiene and Tropical Medicine (LSHTM) (number: 14646), and Marie Stopes International (MSI) ethics committee (number: 003-18). This research project was funded by the Arts & Humanities Research Council (AHRC) (number: AH/R006091/1) and a linked UK Research and Innovation Global Impact Accelerator Award (GIAA). Informed written consent was obtained from all interview and focus group participants in this study.

Patient and public involvement

Neither patients nor the public were involved in the development of the research question, study design or outcome measures. Study participants had some involvement in the conduct of the study as follows. Private providers were involved in recruitment of women seeking abortion services, and factory workers were involved in the production of the intervention through contributing to the scripts, acting in the videos and providing feedback prior to their dissemination. We thank everyone who participated in this research project.

Results

Research context

A summary of sources of information to gather information for the needs assessment is shown in Table 1. Through observation, discussion and the limited available literature we were able to learn about the context and lives of garment factory workers in Phnom Penh.(13) There were several factories in the area each employing several thousand workers, typically working six days per week. Workers sat at sewing machines or did other tasks at individual work stations. Between the noise and the factory floors lay-out, there were limited opportunities to talk among colleagues during working hours. Meal times were when workers gathered in small groups, with different people (different ages and different place of origins) coming together rather than a fixed group of friends. The breaks tended to be short, and people either chatted or looked at their phones while having their meals. In terms of living conditions, workers who came from far away areas rented rooms in apartment blocks nears the factories, sharing with family or other co-workers, often from their same area. Apartments were carved up in different rooms typically rented separately, so space was very limited and privacy of any kind hard to come by. While nearby guest

For peer review only - http://bmjopen.bmj.com/site/about/guidelines.xhtml

houses provided the opportunity for discreet romantic relationships, factory workers tended to be surrounded by other people at all times. Shops around factories catered to the needs of the mostly young, hard-working residents: food and daily necessities shops, mobile phone sellers and service providers, and licensed and unlicensed healthcare providers, from simple drugstores to clinics with consultation rooms and beds. There were no government or NGO clinics in the immediate vicinity of our research area, although some private providers had previously received trainings from NGOs through social marketing or social franchising activities. The factories we visited all had infirmaries for first-aid and minor ailments. Mobile phone shops sold new and used mobile phones, ranging from USD 15 for an old style feature phone to USD 200 for a smartphone. Specialized shops created Facebook accounts on behalf of their clients, did small repairs, downloaded apps and downloaded content such as film and music, cheaper than using contract data to download online content. To complete the general picture of the life of factory workers, it is worth noting that as of January 2019, the minimum monthly wage was USD182 per month,(14) which could increase with overtime or for skilled workers, or be lower if workers did fewer hours or worked for workshops instead of established factories. Our interviewees reported paying an average rent of USD 35 per month (for a shared room) plus about USD5 in utilities. Finally, garment factory workers tended to be more in debt than the average Cambodian (40% of factory worker households are in debt, versus a national rate of 37%).(15)

Reproductive health

We conducted 16 interviews with women seeking abortion from private providers located near the factories and 13 interviews with private providers working in different facilities such as infirmaries, pharmacies and bigger clinics. Findings from these interviews are reported elsewhere.(9) In brief, for health issues during worktime, workers were able to use the factory infirmary, and outside of work the option to use public or private providers. The main role of the infirmaries was to treat minor ailments. Only the largest factory provided contraception. Infirmary staff reported that women would seek consultation elsewhere in the event of an unintended pregnancy.

Factory workers' opportunities for using contraception to prevent pregnancy were constrained by their long working hours, limiting their ability to attend distant clinics with restricted opening hours e.g. for long acting contraception. Pharmacies close to their home

For peer review only - http://bmjopen.bmj.com/site/about/guidelines.xhtml

and workplace were therefore the most practical option for healthcare seeking. There was sensitivity and stigma related to obtaining contraception or MA. Women seeking supplies from drug stores or pharmacies would need to wait until no one else was at the counter or whisper to the provider to achieve any privacy. For young unmarried women privacy in taking visible contraception such as the pill was often essential due to negative social attitudes to sexual activity for unmarried women, yet very difficult to achieve in cramped shared living conditions. Young women either shared a room with other young women or sometimes lived with an older female relative. There was also the suggestion that young women in relationships with older, wealthier men (meeting in nearby guest houses) might be unwilling or unable to insist on condom use. For married women long periods living away from partners meant that contraception was only needed intermittently and unpredictably.

Regarding contraception use in general, women reported receiving information from a variety of sources such as family, friends, healthcare providers, or the media. Women had concerns about contraceptives. Some women reported discontinuing contraception due to experience of side effects such as ‘body heat’ sensation, weight loss due to reduced appetite, vomiting, menstruation changes, skin changes, dizziness and fatigue. Some women had never used contraception due to fear of side-effects including fear of infertility.

Financial constraints or a desire to space pregnancies were the main reported reasons to seek an abortion. In most cases, women obtained information about abortion from family and friends. In a few cases, women went directly to a clinic. None reported seeking information via the internet. Although most private providers were owned by a medical professional such as a doctor or pharmacist, the day-to-day running was often done by a nurse or pharmacist who was a family member or hired staff. Most had undergone formal training but some had learnt on the job or through short training courses. There was limited use of treatment guidelines or protocols. Several providers expressed a wish for more training, including Comprehensive Abortion Care (CAC) training. All of the providers offered short-acting contraceptives and medical abortion. Some larger clinics offered ultrasound, surgical abortion (vacuum aspiration) and had beds for patients. A variety of medical abortion products were available in the private providers around the factories. In some cases someone would request the drugs on behalf of someone else. Information given to women was often verbal, with packaging and the drug information leaflet withheld,

For peer review only - http://bmjopen.bmj.com/site/about/guidelines.xhtml

sometimes upon request of the women to protect their privacy in their homes, but also to prevent clients from knowing the brand name and looking for the drug elsewhere. In the cases where a drug information leaflet was given it was not always in the Khmer language nor had pictorial instructions. Hence women often had to remember the sequence of taking the drugs, side-effects and potential warning signs. Information on post-abortion family planning was variable and follow up was generally in the case of problems rather than routine.

Videos about family planning in Cambodia

Given the observed widespread use of Facebook and Youtube among factory workers, and the preference for receiving information via images, video and voice rather than text that emerged from interviews and observations, we searched Youtube for information on contraception, abortion and medical abortion, using a variety of search terms that reflected both medical and more popular terms. There were few results, and none of the videos we found had been posted by NGOs or government sources, possibly because such videos, although they exist, are not optimized for Khmer language and thus not findable when searching in Khmer. The videos that existed were not professionally produced, often consisting of little more than a slide show or a static image with a voice-over, but they were very descriptive and factual, and were beginning to show views in the thousands and tens of thousands. A detailed analysis of this phase of the research will be reported elsewhere, but our working hypothesis, based on YouTube searches, interviews with factory workers and mobile providers, and the increasing number of mobile internet users in the country, was that the use of the internet to seek health information is just at the beginning for the large number of factory workers who are just starting to go online, and that it will increase significally in the next few years.

Intervention development

We held a mid-point workshop in Cambodia in order to discuss the research findings and their implications. Findings were also discussed in separate meetings with stakeholders in the Cambodian Ministry of Health and other NGOs. Consistent with previous research in Cambodia, we identified concerns or experience of side-effects as an important determinant leading to non-use of effective contraception and subsequent unintended pregnancy.(16)(17) These experiences or concerns were shared by potential users of

For peer review only - http://bmjopen.bmj.com/site/about/guidelines.xhtml

contraception and healthcare providers. Some of the reported side-effects such as ‘body heat’, or ‘weight loss’ (as opposed to weight gain) did not appear to be reported in standard contraception provider guidance or materials developed for potential users. We discussed the possibility that Cambodian women could experience more pronounced hormonal side-effects compared with western women due to differences in size and metabolism. This hormonal ‘mismatch’ theory has been described elsewhere,(18) but it was beyond the scope of our study to explore this systematically. We also discussed similarities and differences between Cambodian womens reports and those of women in the UK talking about hormonal contraception being ‘unnatural’ making them feel ‘out of balance’, which again is not widely raised in materials developed for potential users.(19) An attempt was made to construct a logic model of the problem, as per Intervention Mapping techniques (Figure 1). In this model we considered the health problem to be unintended pregnancy (clearly for women who did not want the pregnancy), and the ‘at-risk’ population to be factory workers with unmet need for contraception. We considered behaviors of the at-risk group and environmental agents, and determinants leading to those behaviors.

A summary of potential interventions is shown in Table 2 using some terminology from the World Health Organization classification of digital health interventions.(20) We felt interventions aimed primarily at providers would be challenging due to the number of different providers, their competing financial and business concerns and the need for sustained effort and follow up, and was beyond the scope of the project. It was apparent that some providers had received training on reproductive health topics such as contraception and abortion from NGOs through social marketing or other programmes. Most providers were interested in more training. We facilitated additional training for those that requested it but it was beyond the scope of this project to provide a comprehensive training package. There was also interest from providers in peer-to-peer support for questions and advise leveraging social and informal ties,(21) such as through social media or instant messaging, potentially moderated by a reproductive health expert from an NGO or department health. There did not seem to be any capacity to do this at scale at the time of the study as it would have required a medium-term investment in human resources and a joint approach to be effective. Marie Stopes Ladies, an MSI global service delivery channel that has recently been started in Cambodia, is utilizing such a strategy, by setting up a

For peer review only - http://bmjopen.bmj.com/site/about/guidelines.xhtml

Facebook group to share information among the service providers.(22) In terms of interventions aimed at factory workers, we considered ways of direct provider-to-client communication, such as enrolling potential users of contraception through private providers or infirmaries, but opted against this due to concerns about maintaining communication, due to the high frequency of phone and/or SIM switching, or potential harm arising from other people listening to messaging, since phones were often accessible to several people aside from their owner (23)(24).

Given the widespread consumption of online videos, we considered that this medium could be the most effective in terms of reach and engagement, so we developed three short 'edutainment' videos about contraception. In addition we adapted three informative videos made by MSI from English to the Khmer language, and also adapted the MSI medical abortion 'Mariprist' instruction leaflet to a simple video format. These videos were not formally evaluated. The three short 'edutainment' videos were aimed at users or potential users of contraception aimed to address personal determinants, particularly knowledge and attitudes, towards contraception. Our objective was to frame contraception in a positive way, whilst acknowledging these frequently experienced and discussed context specific side-effects such as body heat but being careful not to perpetuate them or dismiss them as imagined or unimportant. We felt that videos aimed at the general public that could be watched on mobile phones could be an effective way to deliver an intervention whilst limiting potential for harm. We decided to make three videos because female factory workers are not homogenous. Working from the data we gathered from fieldwork, we constructed two "personas," that is fictional characters that represent characteristics associated with distinct groups of women.(25) The first persona represents married women, possibly with children, interested in spacing out births, and able to discuss contraception and abortion with their husbands. The second represents single women who may or may not be in a stable relationship, and who are interested in information about contraception and medical abortion, but don't necessarily discuss it with their partners.

We wrote a terms of reference and invited local media agencies to pitch ideas. We then worked in collaboration with Phare agency to produce the videos.(11) This involved providing the agency with information on target audience and a visit to garment factory area for a pre-testing workshop with garment factory workers to seek their feedback on

For peer review only - http://bmjopen.bmj.com/site/about/guidelines.xhtml

some prototype videos and opinions on how to further improve the content. During this workshop it was discussed that some of the factory workers would act in one of the videos. There was a two-way process by which the scripts were developed. English language versions of the scripts are shown in Appendix 1. The videos were filmed and Khmer subtitles added and the videos were released sequentially through MSIC's social media communication channels.(26)(27) The first video ("Mother") was aimed at married parous women, showing a conversation between a mother and her children whereby the woman was experiencing side-effects from using contraception with the children providing reassurance in a light hearted way. The key message aimed to be that there is a contraceptive method that will fit with you. This was filmed by the agency in Battambang. The second video ("Love") was aimed at unmarried nulliparous women with the concept of a romance story being watched on a smartphone by a goup of factory workers. The key message aimed to be being in a romantic relationship and still having other life opportunities because of using contraception. The romance scenes were filmed in Battambang and the factory worker scenes were filmed in Phnom Penh. The third video ("Baby") was aimed at married or unmarried women with the concept of a Khmer karaoke-style comedy dance/song with a key message of finding the right method that fits with you and your family.

The videos were posted at one monthly intervals on Facebook and evaluated by Havas Champagne, a Cambodian media company after one month.(12) The videos were boosted on the MSIC Facebook page aimed to engage the target audience, female factory workers and reach the wider public. Geo-targeted ads were used to identify and show content where most factories were located. A click to message campaign was also used to encourage people to send messages to MSIC Facebook page. The videos were placed on the News Feed and Facebook suggested videos feed, and Facebook Watch feed. A shortened version rather than the original baby video was released. Overall, unique reach (number of people who saw a post a least once) was 3,462,176 with 2,839,255 engagements (number of people interacting with the content e.g. like, share, comment, reaction), 7,876,734 video plays, 16,000,000 impressions (number of people who saw a post, but may include multiple views by the same people), and 25,637 click to messages (number of people who click the "Send to Message" button that will lead to MSIC Facebook messenger). An automated response

For peer review only - http://bmjopen.bmj.com/site/about/guidelines.xhtml

was set for those who sent a Facebook message and MSIC counselors provided more comprehensive information regarding the topic they asked about and referred to MSIC clinics or services if required. Key metrics per each video are shown in Table 3. In terms of Click and engagement, the 'Love' video achieved the highest click amount to MSI. Figure 2 shows trends in total calls to the MSIC contact centre and shows the number of Facebook messages sent to MSIC during the project period. Both figures show increases through sept and oct with a decrease afterward corresponding to the timing of release of the 'love' and 'song' videos. Data was not collected from the MSIC contact centre or clinics regarding whether referrals were as a result of any specific content.

Discussion

In this paper we have described the process by which we developed interventions to support reproductive health of garment factory workers in Cambodia, primarily three short 'edutainment' videos about contraceptive methods. Our research identified some challenges that Cambodian garment factory workers experience regarding contraception and abortion. Our findings of unmet need for contraception due social and structural constraints limiting womens opportunity to use contraception and their experience or concerns about side-effects is in keeping with previous literature.(17) Some side-effects reported, such as the experience of 'body heat' with the contraceptive pill appear to be more common in Cambodia compared with elsewhere and possible reasons for this could be further explored. Future interventions should be sensitive to the finding that contraceptive side effects may vary by context, and those critical to non-use may not be recognised in World Health Organization or other global literature and guidelines. Information about family planning often came from friends or family, and mass media campaigns in some cases. Use of private providers would in general result in a bias towards information about short-acting methods as few had the training to provide long-acting methods. Furthermore, providers may not have done formal family planning training and would reinforce myths or concerns about methods. Hence we heard many reports of contraception use and subsequent discontinuation which could result in an unintended pregnancy. We considered that videos portraying contraception in a positive way whilst addressing context specific issues could be an effective way to change attitudes and behaviour regarding contraception use.

For peer review only - http://bmjopen.bmj.com/site/about/guidelines.xhtml

Our three edutainment videos aimed to contain different key messages aimed at both married and unmarried women. By working with a media company, it was possible to incorporate fresh perspectives, and to involve factory workers in the production of the videos. One of the main challenges was maintaining the key messages in the script when working in two languages. The videos were promoted through MSIC's communication channels and key metrics evaluated by an independent company. The videos had a greater reach and number of views if compared other videos on the MSIC Facebook page, but it is difficult to know how much of this was due to the 'boosting' effect. The 'Reach' of the videos was similar, however there were differences in the number of 'Clicks' and 'Engagement'. According to Havas/Champagne this could be explained by the creative and message of the 'Love' video which was more relatable to younger audiences. MSIC saw an increase in calls and messages especially in October, which was the month where they found the highest number of inbound calls and Facebook messages with messages doubled compared to previous month. The videos may have influenced people to directly reach out to MSIC contact centre for more information and led to subsequent use of services. However data was not collected from the MSIC contact centre or clinics regarding whether referrals were as a result of the videos and therefore we cannot assume a causal relationship between our videos and the increased contact centre activity.

In general it can be observed that the videos were less polished or professional in production if compared with other reproductive health videos such as produced by Population Services International Khmer or BBC Media Action.(28)(29) However, they are unique in how they involved and address our targeted population in reflecting a narrative language that is familiar and appreciated and could serve as examples of videos that could be developed and evaluated further. Themes of love and romance in particular could be persued further and we recommend collaboration with creative media companies to contribute fresh ideas. The feedback from Champagne media was to use videos with shorter length and less texts on the thumbnail could improve performance in terms of reach, watch time and higher chance of better audieance retention. It is recommended to focus on mobile phones only, since there was virtually no traffic coming from other devices.

With regards to abortion, we observed that a variety of medical abortion products were widely available from the private providers, with variation in practice as observed

For peer review only - http://bmjopen.bmj.com/site/about/guidelines.xhtml

elsewhere.(6) Witholding the leaflet when selling medical abortion was sometimes done to protect the client, according to the provider, but suggests ‘information asymmetry’ whereby the client is less informed and able to shop around. In any case, women purchasing medical abortion often had to rely on verbal information and remember how to take the product, what to expect and if they should return for follow up. As we were not able to identify any reputable existing videos, in addition to the videos described in this article, we adapted a medical abortion leaflet into a video format which can be viewed on the MSIC Facebook page.(26) This was not formally evaluated in the way the contraception videos were. The impact of providing support for medical abortion in different formats could be evaluated in a further study. In the meantime, our findings on leaflet retention and comprehension by pharmacy clients have contributed to an evidence brief published by Marie Stopes International on supporting self-management of medication abortion from pharmacies.(30)

In this project we did not develop any interventions directly aimed at providers. This was partly because it was difficult to establish the exact status of the providers in terms of current licence, approval to provide certain types of services and quality of medications. Whilst we found varied adherence to best practice, it was encouraging that some providers expressed interest in further training or peer-support groups. Interventions to support or systems to inspect private providers should be explored further.

Strengths of this study include the interdisciplinary approach, collaborations between different academic institutions, an NGO and a media company, and the independent evaluation of the videos. This study has several limitations. Being a qualitative study, the number of participants was relatively small and may not be representative of neither garment factory workers nor providers. Our goal was to reach a good variety of participants, but it is possible that the factories and providers who agreed to participate in the study had better conditions and services compared with other ones. Regarding those seeking abortion services, single women were under-represented in our interviews, and remain a very hard group to reach. Negative experiences of contraception may have been over-reported as many of the women we interviewed had experienced an unintended pregnancy. It is also possible that women’s experiences of contraception, particular side-effects were difficult to translate from Khmer to English, although this was mitigated by having a Cambodian midwife and a linguist on the research team. Intervention mapping provides a rigorous

For peer review only - http://bmjopen.bmj.com/site/about/guidelines.xhtml

detailed step by step approach to developing interventions.(31) In our experience, although we found elements of the approach useful, particularly in conceptualising the problem, other aspects were more challenging to apply. The range of local partners involved were limited to MSI, pharmacists, drug store owners and the factories which combined with the limited time frame and funding for the project constrained our options for the types of intervention we could consider. It was challenging to explain and translate details of intervention mapping across languages, cultures and disciplines working in different paradigms, especially as most of the team were unfamiliar with this approach. A strength of our work was the wide range of disciplines, cultures and perspectives we came from. As a result, our discussions identified similar or overlapping constructs which different people labelled in different ways. In the process of allowing the range of perspectives to contribute in ways that participants felt comfortable we abandoned some of the IM mapping steps which were experienced as too rigid, formulaic and as coming from and using labels from one particular paradigm. We sought to employ participatory approaches in our needs assessment, factory workers views and local MSI staff were involved in developing the concepts for the videos. We obtained feedback from factory workers regarding the concept and content of videos allowing them to inform the process. Some factory workers volunteered to take part in a video. The process resulted in clearer documentation of interventions developed, but alternate interventions might have been developed using the approach applied more strictly or in a different way.

In conclusion, in this paper we describe the development of an intervention to support reproductive health among garment factory workers in Cambodia. These videos could be further improved and additional videos could be developed. More work is required to develop appropriate and effective interventions to support reproductive health of garment factory workers in Cambodia.

List of Abbreviations

AHRC	Arts & Humanities Research Council
BBC	British Broadcasting Corporation
CAC	Comprehensive Abortion Care

For peer review only - http://bmjopen.bmj.com/site/about/guidelines.xhtml

GIAA	Global Impact Accelerator Award
LSHTM	London School of Hygiene and Tropical Medicine
MSI	Marie Stopes International
MSIC	Marie Stopes International Cambodia
NGO	Non Governmental Organisation
UK	United Kingdom
USD	United States Dollar

For peer review only

Declarations

Ethical approval and consent to participate

Ethical approval was obtained from the Cambodia Human Research Ethics committee (number: 094NECHR), the London School of Hygiene and Tropical Medicine (LSHTM) (number: 14646), and Marie Stopes International (MSI) ethics committee (number: 003-18). Informed written consent was obtained from all interview and focus group participants in this study.

Consent for publication

Not applicable

Data sharing statement

The data that support the findings of this study are available on request from the corresponding author [CS] by emailing christopher.smith@lshtm.ac.uk. The data are not publicly available because they contain information that could compromise research participants' privacy and consent.

Competing interests

The authors declare that they have no competing interests

Funding

This study was funded by the Arts & Humanities Research Council (AHRC) (number: AH/R006091/1) and a linked UK Research and Innovation Global Impact Accelerator Award

For peer review only - http://bmjopen.bmj.com/site/about/guidelines.xhtml

(GIAA). The funders had no role in study design collection, management, analysis and data interpretation, writing of the report, and the decision to submit for publication.

Contributor statement

CS contributed the conception and design of the research, data acquisition, data analysis and interpretation, and drafted the manuscript. LS contributed the conception and design of the research, data acquisition, and substantively revised the manuscript. CT contributed the conception and design of the research and substantively revised the manuscript. MM contributed the conception and design of the research, data acquisition, data analysis and interpretation, and substantively revised the manuscript. CF contributed the conception and design of the research and substantively revised the manuscript. JW contributed the conception and design of the research. OA contributed the acquisition of data, read and approved the final manuscript. CM contributed to data acquisition, analysis and interpretation. EO contributed the conception and design of the research, data acquisition, data analysis and interpretation, and drafted the manuscript.

Acknowledgements

We would like to appreciate many clients, factory managers and private providers who contributed to this study. We would also like to thank Marie Stopes International Cambodia for significant cooperation in the entire process of this study. Lastly, we would like to show our gratitude to Professor Tung Rathavy, Director of the National Centre for Maternal and Child Health Centre, for conceptualization and inputs on this study.

References

1. National Institute of Statistics: Directorate General for Health and ICF International. Cambodia Demographic and Health Survey 2014 [Internet]. 2015. Available from: https://dhsprogram.com/pubs/pdf/FR312/FR312.pdf
2. The World Bank In Cambodia. World Development Indicators: Cambodia [Internet]. [cited 2019 Dec 12]. Available from: https://www.worldbank.org/en/country/cambodia
3. Better Factories Cambodia. Better Factories Cambodia: Our Programme [Internet]. 2020 [cited 2020 Aug 31]. Available from: https://betterwork.org/where-we-work/cambodia/bfc-programme/
4. Heng M, Bajracharya A. Garment Sector Health Interventions in Cambodia: A Comprehensive Review. The Evidence Project. Population Council.; 2017.
5. Partnering to Save Lives. Reproductive, Maternal and Neonatal Health Knowledge, Attitudes and Practices among Female Garment Factory Workers in Phnom Penh and Kandal Provinces. 2014.

For peer review only - http://bmjopen.bmj.com/site/about/guidelines.xhtml

6. Petitet PH, Ith L, Cockroft M, Delvaux T. Towards safe abortion access: An exploratory study of medical abortion in Cambodia. Reprod Health Matters. 2015;22(44):47–55.
7. Vonthanak S, Bunsoth M, Sovanthida S, Rathavy T, Kunthea S, Kapp N, et al. A prospective, comparative study of clinical outcomes following clinic-based versus self-use of medical abortion using mifepristol with misoprostol. University of Health Sciences, National Maternal and Child Health Centre, IPAS; 2019.
8. Oreglia E, Ly S, Tijamo C, Ou A, Free C, Smith C. Development of an intervention to support reproductive health of women after seeking medical abortion in Cambodia: Research protocol. JMIR Res Protoc. 2020;9(7):1–12.
9. Masuda C, Oreglia E, Ly S, McLaren M, Free C, Tijamo C, et al. Family planning practices of women working in the Cambodian garment industry: a qualitative study. Contracept Reprod Med. 2020;5(11):1–11.
10. Bartholomew L, Markham C, Ruiter R, Fernandez M, Kok G, Parcel G. Planning Health Promotion Programs: an intervention mapping approach. 4th Ed. Jossey-Bass, editor. Jossey-Bass; 2016.
11. Phare Creative Studio [Internet]. [cited 2019 Dec 12]. Available from: https://pharestudio.org/
12. Havas Champagne [Internet]. [cited 2019 Dec 12]. Available from: https://www.eurocham-cambodia.org/member/276/CHAMPAGNE
13. Chiek C. The Standard of Living of Garment Workers in Cambodia: Its Determinants and Workers' Perception [Internet]. Cambodian Institute for Cooperation and Peace Working Paper No.26. 2008 [cited 2020 Aug 30]. Available from: https://pdfs.semanticscholar.org/f608/a529ae0711be5e7b29970e374ae5b7e1b634.pdf
14. Blomberg M. Mass sackings send chill through Cambodian garment factories [Internet]. Reuters. 2019 [cited 2019 Dec 12]. Available from: https://www.reuters.com/article/us-cambodia-women-garments/mass-sackings-send-chill-through-cambodian-garment-factories-idUSKCN1P41QH
15. ILO. Cambodian Garment and Footwear Sector Bulletin [Internet]. 2018. Available from: https://www.ilo.org/asia/publications/issue-briefs/WCMS_631686/lang--en/index.htm
16. Smith C, Vannak U, Sokhey L, Ngo T, Gold J, Free C. Mobile Technology for Improved Family Planning (MOTIF): the development of a mobile phone-based (mHealth) intervention to support post-abortion family planning (PAFP) in Cambodia. Reprod Health. 2016;13(1):1–8.
17. Petitet P, Desclaux A. Reproductive health and HIV in Cambodia: from anthropology to public health [Internet]. Groupe de Recherche Cultures, Santé, Sociétés, Paul Cézanne Université, Aix Marseille III (France); 2011. Available from: http://hal.archives-ouvertes.fr/docs/00/58/42/95/PDF/Hancart_Desclaux_2010.pdf
18. Alvergne A, Stevens R, Gurmu E. Side effects and the need for secrecy: characterising discontinuation of modern contraception and its causes in Ethiopia using mixed methods. Contracept Reprod Med. 2017;2(1):24.
19. Chang E, Free C. The factors involved in young women's hormonal contraception decisions. A depth interview study. Contraception. 2005;71(6):426–31.
20. World Health Organization. Classification of digital health interventions v1.0 [Internet]. 2018 [cited 2020 Aug 30]. Available from: https://www.who.int/reproductivehealth/publications/mhealth/classification-digital-

For peer review only - http://bmjopen.bmj.com/site/about/guidelines.xhtml

health-interventions/en/
21. Lave J, Wenger E. Situated Learning: Legitimate Peripheral Participation. Cambridge University Press: Cambridge University Press; 1991.
22. Marie Stopes International Cambodia. Marie Stopes Ladies [Internet]. 2020 [cited 2020 Aug 31]. Available from: https://www.mariestopes.org/what-we-do/our-approach/marie-stopes-ladies/
23. Reiss K, Andersen K, Pearson E, Biswas K, Taleb F, Ngo TD, et al. Unintended Consequences of mHealth Interactive Voice Messages Promoting Contraceptive Use After Menstrual Regulation in Bangladesh: Intimate Partner Violence Results From a Randomized Controlled Trial. Glob Heal Sci Pract. 2019;7(3):386–403.
24. Bacchus LJ, Reiss K, Church K, Colombini M, Pearson E, Naved R, et al. Using Digital Technology for Sexual and Reproductive Health : Are Programs Adequately Considering Risk ? 2019;7(4):507–14.
25. Dam R, Teo Y. Personas - A Simple Introduction [Internet]. Interaction Design Foundation. 2019 [cited 2020 Aug 30]. Available from: https://www.interaction-design.org/literature/article/personas-why-and-how-you-should-use-them
26. Marie Stopes International Cambodia. Marie Stopes Cambodia Facebook page [Internet]. 2019 [cited 2019 Dec 12]. Available from: https://www.facebook.com/MarieStopesKh/videos/
27. Marie Stopes International Cambodia. Marie Stopes International Cambodia YouTube channel [Internet]. YouTube. 2020 [cited 2020 Aug 30]. Available from: https://www.youtube.com/channel/UCHIQ85EqjAIyl3tCmuVb0GQ/videos
28. BBC Media Action. BBC Media Action Cambodia [Internet]. 2020 [cited 2020 Aug 30]. Available from: https://www.bbc.co.uk/mediaaction/where-we-work/asia/cambodia
29. PSI. Population Services International Cambodia [Internet]. 2020 [cited 2020 Aug 30]. Available from: https://www.psi.org/country/cambodia/
30. Maries Stopes International. Evidence summary: supporting self-management of MA from pharmacies [Internet]. MSI evidence brief. 2020 [cited 2020 Oct 10]. Available from: https://www.safeaccesshub.org/access/evidence-summary-supporting-self-management-of-ma-from-pharmacies/
31. McCarthy OL, Wazwaz O, Osorio Calderon V, Jado I, Saibov S, Stavridis A, et al. Development of an intervention delivered by mobile phone aimed at decreasing unintended pregnancy among young people in three lower middle income countries. BMC Public Health. 2018;18(1):1–15.

Figure 1: Logic model of the problem

For peer review only

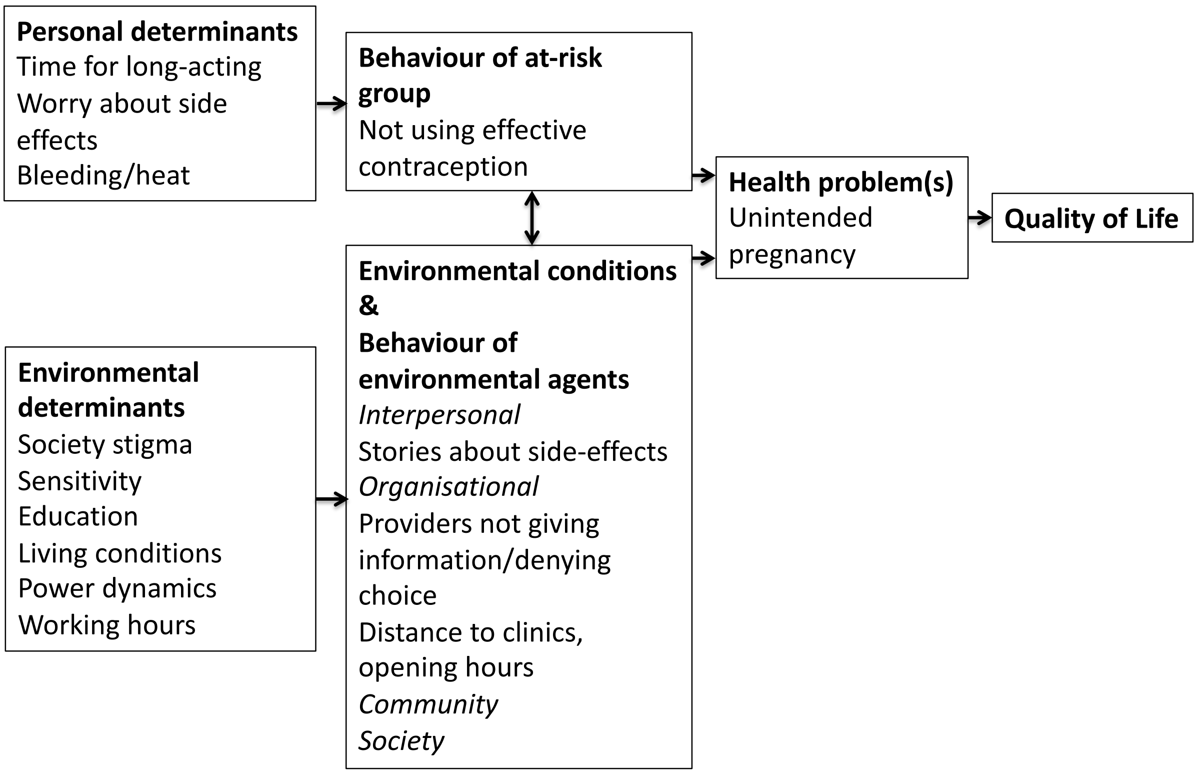

Table 1: Sources of information for the needs assessment

Information domain	Published and grey literature	Observation	Interviews (including discussions)
Factory worker demographics	70% are aged <30 years, half have only primary school education, and most of them have migrated from rural areas away from their family and community support. Higher rate of abortion compared to young women in the national survey, contraception use	Observations of factory workers in and outside of factories consistent with literature	Interviews consistent with literature and observations, although not randomly selected
Factory workers daily life and living conditions outside the factory	Grey literature (NGO reports and newspaper articles) but very limited in detail. Academic literature focused on specific aspects of health and earnings. Limited qualitative work highlighting living conditions (Chansanphors 2008)	Observations in factories (working space, canteen, infirmary, public spaces) , in shops and markets near factories and in 1 worker's home. This provided a useful perspective in the spaces where workers live, what kind of goods and services they have access to, how far they need to go to access specific services.	Formal interviews with 33 female factory workers. "Hanging out" at break times, over meals and at their homes for informal conversations. This allowed us to build rapport, and to contextualize, triangulate and clarify information received from a variety of sources.

For peer review only - http://bmjopen.bmj.com/site/about/guidelines.xhtml

Sources of information for family planning and abortion	Literature and grey literature on contraception and abortion needs and services. No research exists on use of web sources for health/sexual health information in Cambodia.	- Direct observation of online activities did not yield any finding regarding searching for family planning information.<br>- Direct search on YouTube for 'family planning' 'abortion' 'medical abortion' 'contraceptive pill' showed several videos on the topics, some instructional some editorial. All videos had comments, many from 2019 (regardless of when the video was first published), which indicate increasing engagement with online sources to look for family planning information.	Interviews with factory workers indicated a strong reliance on family and friends for information related to contraception and abortion. Medical practitioners were also cited as a source of information, but less influential. Interviewees who were asked directly denied looking for family planning information online, but some said they looked for other health information on Facebook or Youtube.
Family planning providers reproductive health practices	Published literature on family planning in Cambodia	Observation in garment factory infirmaries and private providers	Interviews with 22 providers, including factory nurses, pharmacist, private nurses and doctors.

Table 2: Examples of possible interventions

Example(s)	Intervention classification	Main target	Potential benefits	Potential challenges/disadvantages
Edutainment video about contraception	Targeted client communication (transmit targeted health information to client based on health status or demographics)<br>Goal is to increase awareness, not to instruct on specific details of contraceptive methods	Clients (potential or existing users of contraception, factory workers)	Potential for increased reach<br>Potential to influence attitudes and behaviour about contraception use	Does not provide detailed information about pros and cons of contraceptive method
Provider-to-client communication	Targeted alerts/reminders on mobile phones	Existing clients	Increase in follow-up visits and continuity of care<br>Increase in adherence to instructions	Privacy (if phones are shared or anyway accessible to other people)<br>Frequent changes of numbers and loss of phones make it difficult to have unique phone ID for unique clients

For peer review only - http://bmjopen.bmj.com/site/about/guidelines.xhtml

Provider to provider communication	Increase sharing of best practices in informal private online groups (e.g. Whatsapp and Facebook group chats)	Providers, especially private providers who do not have regular opportunities for professional updates and training	- Provide informal opportunities for sharing best practices and asking for advice<br>- Leverage social and informal ties to create strong 'communities of practice' (Lave & Wenger, 1991)	- Informal groups can be difficult to sustain without participants' buy-in; need a few motivated individuals.<br>- Peer-to-peer information exchanges are not necessarily medically sound, so they could help spread misinformation
Instructional video about contraception methods	On-demand information services to clients	- Existing clients<br>- Potential users already considering a specific method<br>- Providers who need reminding/training about how specific methods work	- Video more attractive format compared with written text, and already a common source of information among targeted audiences.<br>- Can reach clients who are not comfortable or able to go to pharmacies/clinics<br>- comments to the videos can offer insights into frequent questions, and potentially serve as a source of referral for clinics.	Unclear how likely videos are in influencing attitudes and behaviour<br>Rely on being found in the midst of other commercial videos fighting for attention<br>Rely on users having the connectivity to watch the video<br>Requires resources to produce, post, and keep updated<br>Requires resources to potentially monitor and answer comments and questions
Video about abortion	Education about abortion	- Clinic/pharmacy clients<br>- Women at risk of or with unintended pregnancy	- Can reach audiences that are uncomfortable with text and/or with visiting clinics to ask for information<br>- Can offer a medically sound perspective, among propaganda and medically dubious videos currently available online.	- Difficult topic to engage with through an accurate, but accessible and engaging video<br>- Requires significant effort in managing the online presence of such videos (moderation, reliability, findability among competing anti-abortion videos, etc)
Instructional video about medical abortion	Education about MA	- Women with unintended pregnancy	- More accessible alternative to written text for those with low-literacy levels.<br>- Can be easier to access in private than written leaflets.	- Needs to be found online, against existing competing videos that might be less accurate but are ranked higher in search results<br>- Privacy issues, as it remains in search history

For peer review only

For peer review only - http://bmjopen.bmj.com/site/about/guidelines.xhtml

Table 3: Key metrics of the three videos one month after release

	Mother	Love	Baby (shortened)
Video release	13 Aug 2019	Sept 13, 2019	11 Oct 2019
Boost budget	$1,016	$1,016	$1,166
Video plays	2,268,736	2,834,282	2,704,121
Engagement and rate (e.g. likes, shares, comments)	679,591	1,265,398	873,388
Engagement rate	20%	38%	24%
Reach (nationwide)	1,406,274	1,307,140	1,305,075
Cost per reach (CPR) (nationwide)	$0.30	$0.32	$0.32
Click to Action (CTA) (*send message to Marie Stopes)	3,997	23,700	4,728
Cost per click (CPC)	$0.03	$0.01	$0.03

Definitions: Reach is the number of people who saw a post at least once. Engagement is the number of people interacting with the content. Example is like, share, comment, reactions. Click to Action measures the number of people who click the "Send message" button that will lead to MSIC Facebook messenger.

For peer review only

Figure 2: Contact centre total calls and Facebook messages (inbound and outbound)

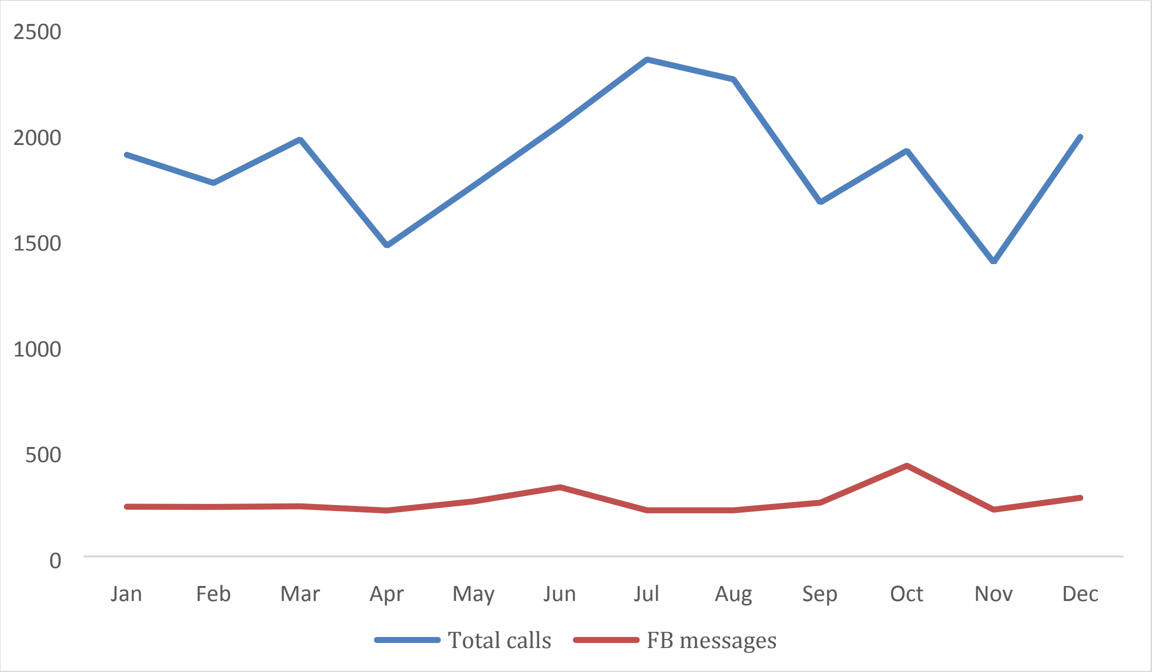

The first video was published in August, second was in September and third was in Oct.

There was a 5-day public holiday in November.

For peer review only

Appendix 1: video scripts

LOVE VIDEO: https://youtu.be/nVOSnsLvrBY	
**Character**	**Script**
BOPHA:	She is not married yet! Hmm... I would be afraid of getting pregnant!
SAMBO:	If I was her, I will use Condom in this situation.
MARDI:	But if you forgot to take the condoms. For me I would use injection every 3 months, so I do not worry about pregnancy anymore! Nobody knows I'm using it. It's easy to enjoy the romance.
LINDA:	I feel more confident by using the pill. My mother used it before she had me. It did not affect her health at all...
BOPHA:	... at least she had a very beautiful daughter!
HEANG:	I chose implant! Here it is! Implants are highly effective up to 3-5 years and discreet. I was not afraid to choose it as my older sister used it before. I will ask a provider to remove it only when I want to have a baby.
BOPHA:	Sorry girls, I have never used anything you talk about. I worry that in a spontaneous situation, I don't have any way to avoid unintended prenancy. Using contraception for protection is better than abortion.
Kunthea:	Hope is not a safe plan.This is my first child. I don't want to have another one right now. We need sometime to save money. You know, why I use IUD. IUD provides protection for up to 10 years long.It is small flexible plastic devide inserted into uterus. and it does not release Hormom like other method and i do not worry about pregnant when my husband back home i still have romance love as a couple on the rice field.
LADY IN RICEFIELD:	What method you choose is your choice. Keep calm and chose the contraceptive methods that fit the most with your life. Do not worry about getting pregnant!
VOICE OVER:	For more information about finding the right method that fits with for your health, or if contraception goes wrong and you need emergency contraception, call Marie Stopes to speak to an adviser<br>012 999 002/098 999 102 . Or find us on FB at https://www.facebook.com/MarieStopesKh

BABY DANCING VIDEO: https://youtu.be/RhmXnc-lvNs	
**Character**	**Script**
Doctor:	If you have any issue, come and talk to us in order to find a solution
Lady:	I feel hot and cold. Do not want to use pill. Even when I use it, don't use it regularly.
Doctor:	If you don't feel good, you should prevent in advance. Don't be careless to have unintended pregnancy. Prevention is better.
Lady:	It seems too early. It is weird for me without husband but using contraception
Doctor:	You have the choice, between injection, implant, IUD and condom. it's simple and easy.
Lady:	I am satisfied taking the pill but I have to know that I have to take it everyday. Thank you doctor for accurate information! Now I have no doubt, less anxiety to use contraception and there are many family planning methods for everybody. You have to think and choose correctly. Don't let yourself have sadness or bad problem because do not use any prevention.
Doctor + lady:	Nothing is easier than prevention. It make you have a happy family and a better future.
Voice over:	For more information about finding the right method that fits with for your health, or if contraception goes wrong and you need emergency contraception, call Marie Stopes to speak to an adviser 012 999 002/098 999102 . Or find us on FB at https://www.facebook.com/MarieStopesKh

MOTHER VIDEO: https://youtu.be/1GzGDI61Ssc	
**Character**	**Script**
YOUNG BROTHER:	What's wrong mum?
MUM:	I just feel dizziness, nausea and hot
YOUNG BROTHER:	Why? Are you sick Mum?

For peer review only - http://bmjopen.bmj.com/site/about/guidelines.xhtml

MUM:	I'm fine, don't worry, I am not sick at all, ...I just take medicine to prevent having another child
SISTER:	Mum wants to say that she took contraceptive' pills
SISTER:	You must take 1 pill every day regularly for it towork. It contains 2 hormones, similar to those that are already found in every woman's body.
YOUNG BROTHER:	Ah...I see. Do you have any side effects Mum?
MUM:	Yes I do son.
SISTER:	Don't worry Mum. The side-effects often go away after 2-3 months. Not everybody gets side effects like you. Do you know, it makes woman have regular periods, and could make you have nice skin and clear acne.
YOUNG BROTHER:	Are there any other methods that more effective and safe?
SISTER:	Well I see 3 more options. Isn't it Mum?
SISTER:	Injections are effective for 3 months. Nobody knows you are using it. You might have some irregular bleeding at first then you might have lighter bleeding. This happens because the uterus lining is not building up. It is normal to disapear over time. It does not affect your health.
SISTER:	If you want a long term family planning Implant is a good choice. It is effective for 3-5 years. It is a small soft plastic pipe. It takes the provider only 5 minutes to insert Implant to put it under arm skin or remove it. And when you want to have another child you just go to see provider to remove it. When it is take out you can have the baby again as normal.
SISTER:	The Intra Uterine Device or IUD is effective for 10 years. It is small and does not release hormones. It stops sperm from meeting the eggs to fertilise. No surgery is required to insert and remove IUD. It makes woman have regular periods.
MUM:	Thank you kids for all your advises!
BABY:	Don't worry Mum! Be confident with your choice for our future. We are here supporting you. Now you know there are other contraception methods for you to choose.
VOICE OVER:	For more information about finding the right method that fits with for your health, or if contraception goes wrong and you need emergency contraception, call Marie Stopes to speak to an adviser<br>012 999 002/098 999102 . Or find us on FB athttps://www.facebook.com/MarieStopesKh

For peer review only

For peer review only - http://bmjopen.bmj.com/site/about/guidelines.xhtml

BMJ Open

Development of an intervention to support reproductive health of garment factory workers in Cambodia: a qualitative study

Journal:	*BMJ Open*
Manuscript ID	bmjopen-2021-049254.R1
Article Type:	Original research
Date Submitted by the Author:	17-Aug-2021
Complete List of Authors:	Smith, Chris; Nagasaki University, Tropical Medicine and Global Health; London School of Hygiene and Tropical Medicine, Clinical Research Department<br>Sokhey, Ly; Marie Stopes International Cambodia<br>Tijamo, Camille Florence; Marie Stopes International Cambodia<br>McLaren, Megan; Marie Stopes International<br>Watkins, Justin; School of Oriental and African Studies (SOAS)<br>Amra, Ou; Marie Stopes International Cambodia<br>Masuda, Chisato; Nagasaki University<br>Oreglia, Elisa; Kings College London
**Primary Subject Heading**:	Reproductive medicine
Secondary Subject Heading:	Qualitative research
Keywords:	Reproductive medicine < GYNAECOLOGY, PUBLIC HEALTH, QUALITATIVE RESEARCH, REPRODUCTIVE MEDICINE

SCHOLARONE™
Manuscripts

I, the Submitting Author has the right to grant and does grant on behalf of all authors of the Work (as defined in the below author licence), an exclusive licence and/or a non-exclusive licence for contributions from authors who are: i) UK Crown employees; ii) where BMJ has agreed a CC-BY licence shall apply, and/or iii) in accordance with the terms applicable for US Federal Government officers or employees acting as part of their official duties; on a worldwide, perpetual, irrevocable, royalty-free basis to BMJ Publishing Group Ltd ("BMJ") its licensees and where the relevant Journal is co-owned by BMJ to the co-owners of the Journal, to publish the Work in this journal and any other BMJ products and to exploit all rights, as set out in our licence.

The Submitting Author accepts and understands that any supply made under these terms is made by BMJ to the Submitting Author unless you are acting as an employee on behalf of your employer or a postgraduate student of an affiliated institution which is paying any applicable article publishing charge ("APC") for Open Access articles. Where the Submitting Author wishes to make the Work available on an Open Access basis (and intends to pay the relevant APC), the terms of reuse of such Open Access shall be governed by a Creative Commons licence – details of these licences and which Creative Commons licence will apply to this Work are set out in our licence referred to above.

Other than as permitted in any relevant BMJ Author's Self Archiving Policies, I confirm this Work has not been accepted for publication elsewhere, is not being considered for publication elsewhere and does not duplicate material already published. I confirm all authors consent to publication of this Work and authorise the granting of this licence.

For peer review only

Title page

Development of an intervention to support reproductive health of garment factory workers in Cambodia: a qualitative study

Authors

Chris Smith[1,2]

Ly Sokhey[3]

Camille Tijamo[3]

Megan McLaren[4]

Caroline Free[5]

Justin Watkins[6]

Ou Amra[3]

Chisato Masuda[1]

Elisa Oreglia[7]

Affiliations

[1] School of Tropical Medicine and Global Health, Nagasaki University, Sakamoto 1-12-4, Nagasaki 852-8523, Japan

[2] Department of Clinical Research, London School of Hygiene and Tropical Medicine, Keppel Street, London WC1E7HT, United Kingdom

[3] Marie Stopes International Cambodia

[4] Marie Stopes International, London, UK

[5] Department of Population Health, London School of Hygiene and Tropical Medicine, Keppel Street, London WC1E7HT, United Kingdom

[6] School of Oriental and African Studies (SOAS)

[7] Kings College London

Corresponding author

Chris Smith

Email: christopher.smith@lshtm.ac.uk

Tel: +81(0)80-8710-5309

School of Tropical Medicine and Global Health, Nagasaki University, Sakamoto 1-12-4, Nagasaki 852-8523, Japan

Trial registration: N/A

For peer review only

For peer review only - http://bmjopen.bmj.com/site/about/guidelines.xhtml

Abstract (300 words)

Objectives: The aim of this study was to describe the development of an intervention to support the reproductive health of garment factory workers in Cambodia.

Design: A qualitative study informed by intervention mapping which included semi-structured interviews and participant observation, followed by intervention development activities including specifying possible behaviour change, designing the intervention, and producing and refining intervention content.

Setting: The research was conducted by a multidisciplinary team with backgrounds in public health, linguistics, digital cultures, and service delivery in a suburb of Phnom Penh where many garment factories cluster.

Participants: Garment factory workers in Cambodia; typically young women aged under 30 years who have migrated from rural areas to Phnom Penh city.

Outcomes: Analysis of reproductive health issues facing garment factory workers and metrics of videos developed.

Results: Our research identified some challenges that Cambodian garment factory workers experience regarding contraception and abortion. Concerns or experience of side-effects were identified as an important determinant leading to non-use of effective contraception and subsequent unintended pregnancy. Financial constraints or a desire to space pregnancies were the main reported reasons to seek an abortion. Information about medical abortion given to women by private providers was often verbal, with packaging and the drug information leaflet withheld. In order to address limitations in the provision of accessible reproductive health information for factory workers, and given their observed widespread use of social media, we decided to make three 'edutainment' videos about family planning. Key social media metrics of the videos were evaluated after one month.

Conclusions: We describe the development of an intervention to support reproductive health among garment factory workers in Cambodia. These videos could be further improved and additional videos could be developed. More work is required to develop appropriate and effective interventions to support reproductive health of garment factory workers in Cambodia.

Keywords: Family planning, contraception, abortion, Cambodia, garment factory workers, intervention development, intervention mapping, videos, behaviour change

For peer review only - http://bmjopen.bmj.com/site/about/guidelines.xhtml

Strengths and limitations of the study

- This study sought to understand the reproductive health issues of a potentially vulnerable population of garment factory workers in Cambodia
- This study was conducted by a multidisciplinary team with backgrounds in public health, linguistics, digital cultures, and service delivery
- We sought to employ participatory approaches in our needs assessment and intervention development
- Being a qualitative study, the number of participants was relatively small and may not be representative of garment factory workers or health providers
- It was challenging to explain and translate details of intervention mapping across languages, cultures, and disciplines working in different paradigms

For peer review only

Development of an intervention to support reproductive health of garment factory workers in Cambodia: a qualitative study

Background

Cambodia has made considerable improvements in economic and health indicators in the last two decades, with much of Cambodia's economic growth attributed to garment exports.(1)(2) In 2015, the estimated number of garment factory workers in Cambodia was estimated to be around 650,000, of whom 85% were female.(3)(4) Garment factory workers are potentially a vulnerable population in terms of support for reproductive health issues and access to services. A study of female factory workers conducted in 2014 reported that 80% were younger than 30 years, half had only primary school education, and most had migrated from rural areas away from their family and community support and did not have adequate access to affordable, high quality reproductive health information and services to meet their diverse needs.(5)

In Cambodia, reproductive health services are available through the public and private sectors, including non-governmental organisations (NGOs) and promoted through traditional and social media, and through community initiatives such as Marie Stopes Ladies.(6)(7)(8) A wide variety of contraception methods are available. Abortion was legalised in 1997 and medical abortion was approved by the Cambodian Ministry of Health in 2009.(9) Previous studies have examined medical abortion provision in clinics and pharmacies. A study in 2015 reported good knowledge of medical abortion among health care providers in clinics and pharmacies in Cambodia.(9) A study of clinical outcomes of medical abortion in 2019 reported no increased need for additional treatment among women seeking medical abortion from pharmacies compared with clinics, but that women were less likely to feel prepared for what happened after taking the pills or to be offered contraceptive methods when getting medical abortion pills. Users of the different services varied with 26% of women in the pharmacy group being garment factory workers compared with 9% in the clinic group.(10)

This current study was conceived based on the prior observation that many independent private healthcare providers are located in the vicinity of garment factories, but not much is known about what kind of reproductive health services they provide, particularly medical

For peer review only - http://bmjopen.bmj.com/site/about/guidelines.xhtml

abortion and contraception, and the experience of clients who visit them. The project is a collaboration between Marie Stopes International Cambodia (MSIC) and three universities in the United Kingdom. The study protocol and description of family planning practices are described elsewhere.(11)(12) The aim of this paper is to describe the development of an intervention to support the reproductive health of garment factory workers in Cambodia.

Methods

This qualitative study was conducted during January 2018 to December 2019 in a suburb of Phnom Penh where many garment factories cluster, with the co-operation of three factories and a number of private healthcare providers. We were a multidisciplinary and multinational team (Cambodian and UK-based researchers) with backgrounds in public health, linguistics, digital cultures (that is, the study of how different people and groups engage with digital technologies), and service delivery. The research was conducted by different members of the research team at different times, as illustrated in Figure 1. Each member focused on their area of expertise and methods, while participating in data gathering done by other members. Findings from the different phases and approaches informed subsequent data gathering, in an iterative process aimed at understanding the wider context of medical abortion and contraception first (needs assessment), and develop possible interventions later. Research methods included general and participant observation, semi-structured interviews and periodic meetings in the UK and Cambodia to discuss findings.(11)

The methods were informed by intervention mapping methods,(13) but in a more holistic manner, which took into consideration the different disciplines involved. We aimed to conduct a needs assessment followed by intervention development activities including specifying possible behaviour change targets, designing the intervention and producing and refining intervention content. We transcribed and translated all interviews, and coded them both deductively, utilizing codes derived from the structure of the intervention mapping needs assessment process, and inductively, using codes that emerged from the more open-ended methods, including observations and semi-structured interviews with factory workers. At least two different researchers coded each interview, one deductively and one inductively. The end result was not a pure application of intervention mapping but rather a modified approach to it.

For peer review only - http://bmjopen.bmj.com/site/about/guidelines.xhtml

We began with a short ethnographic study of female factory workers' lives, living conditions, work places, and digital technology use, in order to learn about the wider context of choices regarding reproductive health. We selected three different factories with which our local partners had an existing relation, and obtained their permission to visit the factories during working hours to chat with workers. We introduced ourselves as researchers to the factory workers, and were not accompanied by factory representatives during such interactions. We joined several women, individually or in small groups, during their lunch breaks in factory grounds, chatting about their experiences and getting a sense of who they were friends with, what they discussed during breaks, how they found their current jobs and what their future plans were. We also shared meals with them in nearby restaurants after working hours and at weekends, and were able to visit some of their homes. After an initial period of what ethnographers call "hanging out", that is unstructured time spent with research participants doing whatever they do, in order to become familiar with their lives and to give them time to get used to our presence,(14) we conducted 17 semi-structured interviews, using snowball sampling, that focused primarily on their mobile phone use, to understand what sites/apps they used, what content they consumed online and off, and whether they searched for specific information. Some of the interviews covered healthcare and health-related topics. We also did three semi-structured interviews with owners of mobile phone shops and service providers near the factories, to triangulate what we learnt from the interviews about mobile phone use.

At the same time, we observed the practice of and interviewed garment factory infirmary workers and a variety of private healthcare providers ranging from simple drug stores to larger clinics, in order to learn about garment factory workers' use of reproductive health services. In this phase of research, we conducted 16 interviews with women seeking abortion from private providers located near the factories, focusing on reasons for seeking abortion services and their experiences, and 13 interviews with private providers working in different facilities such as infirmaries, pharmacies and bigger clinics, to understand practices around reproductive health, especially with regards to contraception and abortion provision (Supplementary File 1). Findings from these interviews are reported elsewhere.(12)

During the course of the project we organized several meetings to share and discuss behaviours observed in research that could feasibly be targeted and potential interventions

For peer review only - http://bmjopen.bmj.com/site/about/guidelines.xhtml

resulting from the needs assessment activities, and then design, produce and refine a pilot intervention. In order to address limitations in the provision of accessible contraceptive information and information materials for factory workers in general and specifically at the time of seeking medical abortion we decided to make three 'edutainment' videos about family planning and one instructional video about how to take MA correctly. We collaborated with a Cambodian media company (Phare) and during the process sought input from garment factory workers in a pre-testing workshop.(15) The videos were designed to target barriers to contraception use that were identified in the needs assessment, ethnographic and interviews studies.(12) The videos were released through MSIC's communication channels and their impact was evaluated by an independent digital media company (Havas Champagne media).(16)

Ethics

Ethical approval was obtained from the Cambodia National Ethics Committee for Health Research (NECHR) (number: 094NECHR), the London School of Hygiene and Tropical Medicine (LSHTM) (number: 14646), and Marie Stopes International (MSI) ethics committee (number: 003-18). This research project was funded by the Arts & Humanities Research Council (AHRC) (number: AH/R006091/1) and a linked UK Research and Innovation Global Impact Accelerator Award (GIAA). Informed written consent was obtained from all interview and focus group participants in this study.

Patient and public involvement

Neither patients nor the public were involved in the development of the research question, study design or outcome measures. Study participants had some involvement in the conduct of the study as follows: private providers were involved in recruitment of women seeking abortion services, and factory workers were involved in the production of the intervention through contributing to the scripts, acting in the videos and providing feedback prior to their dissemination. We thank everyone who participated in this research project.

Results

Research context

For peer review only - http://bmjopen.bmj.com/site/about/guidelines.xhtml

A summary of sources of information to gather information for the needs assessment is shown in Table 1. Through observation, discussion and the limited available literature we were able to learn about the context and lives of garment factory workers in Phnom Penh.(17) There were several factories in the area each employing several thousand workers, typically working six days per week. Workers sat at sewing machines or did other tasks at individual work stations. Between the noise and the factory floors lay-out, there were limited opportunities to talk among colleagues during working hours. Meal times were when workers gathered in small groups, with different people (different ages and different places of origins) coming together rather than a fixed group of friends. The breaks tended to be short, and people either chatted or looked at their phones while having their meals. In terms of living conditions, workers who came from far away areas rented rooms in apartment blocks near the factories, sharing with family or other co-workers, often from their same area. Apartments were carved up in different rooms typically rented separately, so space was very limited and privacy of any kind hard to come by. While nearby guest houses provided the opportunity for discreet romantic relationships, factory workers tended to be surrounded by other people at all times. Shops around factories catered to the needs of the mostly young, hard-working residents: food and daily necessities shops, mobile phone sellers and service providers, and licensed and unlicensed healthcare providers, from simple drugstores to clinics with consultation rooms and beds. There were no government or NGO clinics in the immediate vicinity of our research area, although some private providers had previously received trainings from NGOs through social marketing or social franchising activities. The factories we visited all had infirmaries for first-aid and minor ailments. Mobile phone shops sold new and used mobile phones, ranging from USD 15 for an old style feature phone to USD 200 for a smartphone. Specialized shops created Facebook accounts on behalf of their clients, did small repairs, downloaded apps and content such as film and music, cheaper than using contract data to download online content. To complete the general picture of the life of factory workers, it is worth noting that as of January 2019, the minimum monthly wage was USD182 per month,(18) which could increase with overtime or for skilled workers, or be lower if workers did fewer hours or worked for workshops instead of established factories. Our interviewees reported paying an average rent of USD 35 per month (for a shared room) plus about USD 5 in utilities. Finally, garment factory workers

For peer review only - http://bmjopen.bmj.com/site/about/guidelines.xhtml

tended to be more in debt than the average Cambodian (40% of factory worker households were reported to be in debt, versus a national rate of 37%).(19)

Reproductive health

The interviews with women seeking abortion from private providers, and with the private providers showed that in brief, for health issues during worktime, workers were able to use the factory infirmary, and outside of work the option to use public or private providers. The main role of the infirmaries was to treat minor ailments. Only the largest factory provided contraception. Infirmary staff reported that women would seek consultation elsewhere in the event of an unintended pregnancy.

Factory workers' opportunities for using contraception to prevent pregnancy were constrained by their long working hours, limiting their ability to attend distant clinics with restricted opening hours e.g. for long-acting contraception. Pharmacies close to their home and workplace were therefore the most practical option for healthcare seeking but there was sensitivity and stigma related to obtaining contraception or MA near where women lived and worked. Women seeking supplies from drug stores or pharmacies would try to go to places far from where they lived to avoid being recognised, or would need to wait until no one else was at the counter or whisper to the provider to achieve any privacy. Young, unmarried women sought privacy in both buying and taking contraception such as the pill because of negative social attitudes towards sexual activity for unmarried women, yet privacy was very difficult to achieve in cramped and shared living conditions, and in small neighbourhoods where everybody knows everybody. Young women either shared a room with other young women or sometimes lived with an older female relative. There was also the suggestion that young women in relationships with older, wealthier men (meeting in nearby guest houses) might be unwilling or unable to insist on condom use. For married women long periods living away from partners meant that contraception was only needed intermittently and unpredictably.

Regarding contraception use in general, women reported receiving information from a variety of sources such as family, friends, healthcare providers, or the media. Women had concerns about contraceptives. Some women reported discontinuing contraception due to experience of side effects such as 'body heat' sensation, weight loss due to reduced

For peer review only - http://bmjopen.bmj.com/site/about/guidelines.xhtml

appetite, vomiting, menstruation changes, skin changes, dizziness and fatigue. Some women had never used contraception due to fear of side-effects including fear of infertility.

Financial constraints or a desire to space pregnancies were the main reported reasons to seek an abortion. In most cases, women obtained information about abortion from family and friends. In a few cases, women went directly to a clinic. None reported seeking information via the internet. Although most private providers were owned by a medical professional such as a doctor or pharmacist, the day-to-day running was often done by a nurse or pharmacist who was a family member or hired staff. Most had undergone formal training but some had learnt on the job or through short training courses. There was limited use of treatment guidelines or protocols. Several providers expressed a wish for more training, including Comprehensive Abortion Care (CAC) training. All of the providers offered short-acting contraceptives and medical abortion. Some larger clinics offered ultrasound, surgical abortion (vacuum aspiration) and had beds for patients. A variety of medical abortion products were available in the private providers around the factories. In some cases someone would request the drugs on behalf of another person. Information given to women was often verbal, with packaging and the drug information leaflet withheld, sometimes upon request of the women to protect their privacy at home, but also to prevent clients from knowing the brand name and looking for the drug elsewhere. In the cases where a drug information leaflet was given it was not always in the Khmer language nor had pictorial instructions. Hence women often had to remember the sequence of taking the drugs, side-effects and potential warning signs. Information on post-abortion family planning was variable and follow up was generally in the case of problems rather than routine.

Videos about family planning in Cambodia

Given the observed widespread use of Facebook and Youtube among factory workers, and the preference for receiving information via images, video and voice rather than text that emerged from interviews and observations, we searched Youtube for information on contraception, abortion and medical abortion, using a variety of search terms that reflected both medical and more popular terms. There were few results, and none of the videos we found had been posted by NGOs or government sources, possibly because such videos, although they exist, are not optimized for Khmer language and thus not findable when

For peer review only - http://bmjopen.bmj.com/site/about/guidelines.xhtml

searching in Khmer. The videos that existed were not professionally produced, often consisting of little more than a slide show or a static image with a voice-over, but they were very descriptive and factual, and were beginning to show views in the thousands and tens of thousands. A detailed analysis of this phase of the research will be reported elsewhere, but our working hypothesis, based on YouTube searches, interviews with factory workers and mobile providers, and the increasing number of mobile internet and social media users in the country, estimated to have reached 12 million over a population of 16.8 million in January 2021,(20) was that the use of the internet to seek health information is just at the beginning for the large number of factory workers who are just starting to go online, and that it will increase significantly in the next few years.

Intervention development

We held a mid-point workshop in Cambodia in order to discuss the research findings and their implications. Findings were also discussed in separate meetings with stakeholders in the Cambodian Ministry of Health and other NGOs. Consistent with previous research in Cambodia, we identified concerns or experience of side-effects as an important determinant leading to non-use of effective contraception and subsequent unintended pregnancy.(21)(22) These experiences or concerns were shared by potential users of contraception and healthcare providers. Some of the reported side-effects such as 'body heat', or 'weight loss' (as opposed to weight gain) did not appear to be reported in standard contraception provider guidance or materials developed for potential users. We discussed the possibility that Cambodian women could experience more pronounced hormonal side-effects compared with western women due to differences in size and metabolism. This hormonal 'mismatch' theory has been described elsewhere,(23) but it was beyond the scope of our study to explore this systematically. We also discussed similarities and differences between Cambodian womens reports and those of women in the UK talking about hormonal contraception being 'unnatural' making them feel 'out of balance', which again is not widely raised in materials developed for potential users.(24) An attempt was made to construct a logic model of the problem, as per Intervention Mapping techniques (Figure 2). In this model we considered the health problem to be unintended pregnancy (clearly for women who did not want the pregnancy), and the 'at-risk' population to be

For peer review only - http://bmjopen.bmj.com/site/about/guidelines.xhtml

factory workers with unmet need for contraception. We considered behaviors of the at-risk group and environmental agents, and determinants leading to those behaviors.

A summary of potential interventions is shown in Table 2 using some terminology from the World Health Organization classification of digital health interventions.(25) We felt interventions aimed primarily at providers would be challenging due to the number of different providers, their competing financial and business concerns and the need for sustained effort and follow up, and was beyond the scope of the project. It was apparent that some providers had received training on reproductive health topics such as contraception and abortion from NGOs through social marketing or other programmes. Most providers were interested in more training. We facilitated additional training for those that requested it but it was beyond the scope of this project to provide a comprehensive training package. There was also interest from providers in peer-to-peer support for questions and advise leveraging social and informal ties,(26) such as through social media or instant messaging, potentially moderated by a reproductive health expert from an NGO or department health. There did not seem to be any capacity to do this at scale at the time of the study as it would have required a medium-term investment in human resources and a joint approach to be effective. Marie Stopes Ladies, an MSI global service delivery channel that has recently been started in Cambodia, is utilizing such a strategy, by setting up a Facebook group to share information among the service providers.(8) In terms of interventions aimed at factory workers, we considered ways of direct provider-to-client communication, such as enrolling potential users of contraception through private providers or infirmaries, but opted against this due to concerns about maintaining communication, due to the high frequency of phone and/or SIM switching, or potential harm arising from other people listening to messaging, since phones were often accessible to several people aside from their owner (27)(28).

Given the widespread consumption of online videos, we considered that this medium could be the most effective in terms of reach and engagement, so we developed three short 'edutainment' videos about contraception. In addition we adapted three informative videos made by MSI from English to the Khmer language, and adapted the MSI medical abortion 'Mariprist' instruction leaflet to a simple video format. These videos were not formally evaluated. The three short 'edutainment' videos were aimed at users or potential users of

For peer review only - http://bmjopen.bmj.com/site/about/guidelines.xhtml

contraception aimed to address personal determinants, particularly knowledge and attitudes, towards contraception. Our objective was to frame contraception in a positive way, whilst acknowledging these frequently experienced and discussed context specific side-effects such as body heat but being careful not to perpetuate them or dismiss them as imagined or unimportant. We felt that videos aimed at the general public that could be watched on mobile phones could be an effective way to deliver an intervention whilst limiting potential for harm. We decided to make three videos because female factory workers are not homogenous. Working from the data we gathered from fieldwork, we constructed two "personas," that is fictional characters that represent characteristics associated with distinct groups of women.(29) The first persona represents married women, possibly with children, interested in spacing out births, and able to discuss contraception and abortion with their husbands. The second represents single women who may or may not be in a stable relationship, and who are interested in information about contraception and medical abortion, but don't necessarily discuss it with their partners.

We wrote a terms of reference and invited local media agencies to pitch ideas. We then worked in collaboration with Phare agency to produce the videos.(15) This involved providing the agency with information on target audience and a visit to garment factory area for a pre-testing workshop with garment factory workers to seek their feedback on some prototype videos and opinions on how to further improve the content. During this workshop it was discussed that some of the factory workers would act in one of the videos. There was a two-way process by which the scripts were developed. English language versions of the scripts are shown in Supplementary File 2. The videos were filmed and Khmer subtitles added and the videos were released sequentially through MSIC's social media communication channels.(30)(31) MSIC social media followers have grown significantly since 2019, so posting the videos on the MSIC Facebook and Youtube pages allowed us to reach a much broader audience than simply trying to distribute them among the factory workers we knew. The first video ("Mother") was aimed at married parous women, showing a conversation between a mother and her children whereby the woman was experiencing side-effects from using contraception with the children providing reassurance in a light hearted way. The key message aimed to be that there is a contraceptive method that will fit with you. This was filmed by the agency in Battambang.

For peer review only - http://bmjopen.bmj.com/site/about/guidelines.xhtml

The second video ("Love") was aimed at unmarried nulliparous women with the concept of a romance story being watched on a smartphone by a goup of factory workers. The key message aimed to be being in a romantic relationship and still having other life opportunities because of using contraception. The romance scenes were filmed in Battambang and the factory worker scenes were filmed in Phnom Penh. The third video ("Baby") was aimed at married or unmarried women with the concept of a Khmer karaoke-style comedy dance/song with a key message of finding the right method that fits with you and your family. We focused on these three topics as they emerged as the most important concerns for our diverse research participants in the qualitative research phase.

The videos were posted at one monthly intervals on Facebook and evaluated by Havas Champagne, a Cambodian media company after one month.(16) The videos were boosted on the MSIC Facebook page aimed to engage the target audience, female factory workers and reach the wider public. Geo-targeted ads were used to identify and show content where most factories were located. A click to message campaign was also used to encourage people to send messages to MSIC Facebook page. The videos were placed on the News Feed and Facebook suggested videos feed, and Facebook Watch feed. A shortened version rather than the original baby video was released. Overall, unique reach (number of people who saw a post a least once) was 3,462,176 with 2,839,255 engagements (number of people interacting with the content e.g. like, share, comment, reaction), 7,876,734 video plays, 16,000,000 impressions (number of people who saw a post, but may include multiple views by the same people), and 25,637 click to messages (number of people who click the "Send to Message" button that will lead to MSIC Facebook messenger). An automated response was set for those who sent a Facebook message and MSIC counselors provided more comprehensive information regarding the topic they asked about and referred to MSIC clinics or services if required. Key metrics per each video are shown in Table 3. In terms of Click and engagement, the 'Love' video achieved the highest click to messages. Figure 3 shows trends in total calls to the MSIC contact centre and shows the number of Facebook messages sent to MSIC during the project period. Both figures show increases through September and October corresponding to the timing of release of the 'Love' and 'Baby' videos. Data was not collected from the MSIC contact centre or clinics regarding whether referrals were as a result of any specific content.

For peer review only - http://bmjopen.bmj.com/site/about/guidelines.xhtml

Discussion

In this paper we have described the process by which we developed interventions to support reproductive health of garment factory workers in Cambodia, primarily three short 'edutainment' videos about contraceptive methods. Our research identified some challenges that Cambodian garment factory workers experience regarding contraception and abortion. Our findings of unmet need for contraception due social and structural constraints limiting womens opportunity to use contraception and their experience or concerns about side-effects is in keeping with previous literature.(22) Some side-effects reported, such as the experience of 'body heat' with the contraceptive pill appear to be more common in Cambodia compared with elsewhere and possible reasons for this could be further explored. Future interventions should be sensitive to the finding that contraceptive side effects may vary by context, and those critical to non-use may not be recognised in World Health Organization or other global literature and guidelines. Information about family planning often came from friends or family, and mass media campaigns in some cases. Use of private providers would in general result in a bias towards information about short-acting methods as few had the training to provide long-acting methods. Furthermore, providers may not have received formal family planning training and would reinforce myths or concerns about methods. Hence we heard many reports of contraception use and subsequent discontinuation which could result in an unintended pregnancy. We considered that videos portraying contraception in a positive way whilst addressing context specific issues could be an effective way to change attitudes and behaviour regarding contraception use.

Our three edutainment videos aimed to contain different key messages aimed at both married and unmarried women. By working with a media company, it was possible to incorporate fresh perspectives, and to involve factory workers in the production of the videos. One of the main challenges was maintaining the key messages in the script when working in two languages. The videos were promoted through MSIC's communication channels and key metrics evaluated by an independent company. The videos had a greater reach and number of views if compared other videos on the MSIC Facebook page, but it is difficult to know how much of this was due to the 'boosting' effect. The 'Reach' of the videos was similar, however there were differences in the number of 'Clicks' and

For peer review only - http://bmjopen.bmj.com/site/about/guidelines.xhtml

'Engagement'. According to Havas/Champagne this could be explained by the creative and message of the 'Love' video which was more relatable to younger audiences. MSIC saw an increase of inbound calls and Facebook messages especially in October, with messages doubled compared to previous month. The videos may have influenced people to directly reach out to MSIC contact centre for more information and led to subsequent use of services. However data was not collected from the MSIC contact centre or clinics regarding whether referrals were as a result of the videos and therefore we cannot assume a causal relationship between our videos and the increased contact centre activity.

In general it can be observed that the videos were less polished or professional in production if compared with other reproductive health videos such as produced by Population Services International Khmer or BBC Media Action.(7)(6) However, to our knowledge they are unique in how they involved and address our targeted population in reflecting a narrative language that is familiar and appreciated and could serve as examples of videos that could be developed and evaluated further. Themes of love and romance in particular could be persued further and we recommend collaboration with creative media companies to contribute fresh ideas. The feedback from Havas/Champagne media was to use videos with shorter length and less texts on the thumbnail could improve performance in terms of reach, watch time and higher chance of better audieance retention. It is recommended to focus on mobile phones only, since there was virtually no traffic coming from other devices.

With regards to abortion, we observed that a variety of medical abortion products were widely available from the private providers, with variation in practice as observed elsewhere.(9) Witholding the leaflet when selling medical abortion was sometimes done to protect the client, according to the provider, but suggests 'information asymmetry' whereby the client is less informed and able to shop around. In any case, women purchasing medical abortion often had to rely on verbal information and remember how to take the product, what to expect and if they should return for follow up. As we were not able to identify any reputable existing videos, in addition to the videos described in this article, we adapted a medical abortion leaflet into a video format which can be viewed on the MSIC Facebook page.(30) This was not formally evaluated in the way the contraception videos were. The impact of providing support for medical abortion in different formats could be evaluated in

For peer review only - http://bmjopen.bmj.com/site/about/guidelines.xhtml

a further study. In the meantime, our findings on leaflet retention and comprehension by pharmacy clients have contributed to an evidence brief published by MSI on supporting self-management of medication abortion from pharmacies.(32)

In this project we did not develop any interventions directly aimed at providers. This was partly because it was difficult to establish the exact status of the providers in terms of current licence, approval to provide certain types of services and quality of medications. Whilst we found varied adherence to best practice, it was encouraging that some providers expressed interest in further training or peer-support groups. Interventions to support or systems to inspect private providers should be explored further.

Strengths of this study include the interdisciplinary approach, collaborations between different academic institutions, an NGO and a media company, and the independent evaluation of the videos. This study has several limitations. Being a qualitative study, the number of participants was relatively small and is not representative of garment factory workers nor providers. Our goal was to reach a good variety of participants, but it is possible that the factories and providers who agreed to participate in the study had better conditions and services compared with other ones. Regarding those seeking abortion services, single women were under-represented in our interviews, and remain a very hard group to reach. Negative experiences of contraception may have been over-reported as many of the women we interviewed had experienced an unintended pregnancy. It is also possible that women's experiences of contraception, particular side-effects were difficult to translate from Khmer to English, although this was mitigated by having a Cambodian midwife and a linguist on the research team. Intervention mapping provides a rigorous detailed step by step approach to developing interventions.(33) In our experience, although we found elements of the approach useful, particularly in conceptualising the problem, other aspects were more challenging to apply. The range of local partners involved were limited to MSI, pharmacists, drug store owners and the factories which combined with the limited time frame and funding for the project constrained our options for the types of intervention we could consider. It was challenging to explain and translate details of intervention mapping across languages, cultures and disciplines working in different paradigms, especially as most of the team were unfamiliar with this approach. A strength of our work was the wide range of disciplines, cultures and perspectives we came from. As a result, our discussions identified

For peer review only - http://bmjopen.bmj.com/site/about/guidelines.xhtml

similar or overlapping constructs which different people labelled in different ways. In the process of allowing the range of perspectives to contribute in ways that participants felt comfortable we abandoned some of the IM mapping steps which were experienced as too rigid, formulaic and as coming from and using labels from one particular paradigm. We sought to employ participatory approaches in our needs assessment, so factory workers' views and local MSI staff were involved in developing the concepts for the videos. We obtained feedback from factory workers regarding the concept and content of videos allowing them to inform the process. Some factory workers volunteered to take part in a video. The process resulted in clearer documentation of interventions developed, but alternate interventions might have been developed using the approach applied more strictly or in a different way.

In conclusion, we describe the development of three 'edutainment' videos to address limitations in the provision of accessible reproductive health information and support use of family planning among garment factory workers in Cambodia. Key social media metrics of the videos were evaluated after one month. These videos could be further improved and additional videos could be developed. More work is required to develop appropriate and effective interventions to support reproductive health of garment factory workers in Cambodia.

List of Abbreviations

AHRC	Arts & Humanities Research Council
BBC	British Broadcasting Corporation
CAC	Comprehensive Abortion Care
GIAA	Global Impact Accelerator Award
LSHTM	London School of Hygiene and Tropical Medicine
MSI	Marie Stopes International
MSIC	Marie Stopes International Cambodia
NGO	Non Governmental Organisation

For peer review only

SIM	Subscriber Identity Module
UK	United Kingdom
USD	United States Dollar

Declarations

Ethical approval and consent to participate

Ethical approval was obtained from the Cambodia Human Research Ethics committee (number: 094NECHR), the London School of Hygiene and Tropical Medicine (LSHTM) (number: 14646), and Marie Stopes International (MSI) ethics committee (number: 003-18). Informed written consent was obtained from all interview and focus group participants in this study.

Consent for publication

Not applicable

Data sharing statement

The data that support the findings of this study are available on request from the corresponding author [CS] by emailing christopher.smith@lshtm.ac.uk. The data are not publicly available because they contain information that could compromise research participants' privacy and consent.

Competing interests

The authors declare that they have no competing interests

Funding

This study was funded by the Arts & Humanities Research Council (AHRC) (number: AH/R006091/1) and a linked UK Research and Innovation Global Impact Accelerator Award (GIAA). The funders had no role in study design collection, management, analysis and data interpretation, writing of the report, and the decision to submit for publication.

Contributor statement

CS contributed the conception and design of the research, data acquisition, data analysis and interpretation, and drafted the manuscript. LS contributed the conception and design of

For peer review only

the research, data acquisition, and substantively revised the manuscript. CT contributed the conception and design of the research and substantively revised the manuscript. MM contributed the conception and design of the research, data acquisition, data analysis and interpretation, and substantively revised the manuscript. CF contributed the conception and design of the research and substantively revised the manuscript. JW contributed the conception and design of the research. OA contributed the acquisition of data, read and approved the final manuscript. CM contributed to data acquisition, analysis and interpretation. EO contributed the conception and design of the research, data acquisition, data analysis and interpretation, and drafted the manuscript.

Acknowledgements

We would like to appreciate many clients, factory managers and private providers who contributed to this study. We would also like to thank Marie Stopes International Cambodia for significant cooperation in the entire process of this study. Lastly, we would like to show our gratitude to Professor Tung Rathavy, Director of the National Centre for Maternal and Child Health Centre, for conceptualization and inputs on this study.

References

1. National Institute of Statistics: Directorate General for Health and ICF International. Cambodia Demographic and Health Survey 2014 [Internet]. 2015. Available from: https://dhsprogram.com/pubs/pdf/FR312/FR312.pdf
2. The World Bank In Cambodia. World Development Indicators: Cambodia [Internet]. [cited 2019 Dec 12]. Available from: https://www.worldbank.org/en/country/cambodia
3. Better Factories Cambodia. Better Factories Cambodia: Our Programme [Internet]. 2020 [cited 2020 Aug 31]. Available from: https://betterwork.org/where-we-work/cambodia/bfc-programme/
4. Heng M, Bajracharya A. Garment Sector Health Interventions in Cambodia: A Comprehensive Review. The Evidence Project. Population Council.; 2017.
5. Partnering to Save Lives. Reproductive, Maternal and Neonatal Health Knowledge, Attitudes and Practices among Female Garment Factory Workers in Phnom Penh and Kandal Provinces. 2014.
6. PSI. Population Services International Cambodia [Internet]. 2020 [cited 2020 Aug 30]. Available from: https://www.psi.org/country/cambodia/
7. BBC Media Action. BBC Media Action Cambodia [Internet]. 2020 [cited 2020 Aug 30]. Available from: https://www.bbc.co.uk/mediaaction/where-we-work/asia/cambodia
8. Marie Stopes International Cambodia. Marie Stopes Ladies [Internet]. 2020 [cited 2020 Aug 31]. Available from: https://www.mariestopes.org/what-we-do/our-approach/marie-stopes-ladies/
9. Petitet PH, Ith L, Cockroft M, Delvaux T. Towards safe abortion access: An exploratory

study of medical abortion in Cambodia. Reprod Health Matters. 2015;22(44):47–55.

10. Vonthanak S, Bunsoth M, Sovanthida S, Rathavy T, Kunthea S, Kapp N, et al. A prospective, comparative study of clinical outcomes following clinic-based versus self-use of medical abortion using mifepristol with misoprostol. University of Health Sciences, National Maternal and Child Health Centre, IPAS; 2019.

11. Oreglia E, Ly S, Tijamo C, Ou A, Free C, Smith C. Development of an intervention to support reproductive health of women after seeking medical abortion in Cambodia: Research protocol. JMIR Res Protoc. 2020;9(7):1–12.

12. Masuda C, Oreglia E, Ly S, McLaren M, Free C, Tijamo C, et al. Family planning practices of women working in the Cambodian garment industry: a qualitative study. Contracept Reprod Med. 2020;5(11):1–11.

13. Bartholomew L, Markham C, Ruiter R, Fernandez M, Kok G, Parcel G. Planning Health Promotion Programs: an intervention mapping approach. 4th Ed. Jossey-Bass, editor. Jossey-Bass; 2016.

14. Salvador T, Genevieve B, Anderson K. Design ethnography. Des Manag J. 1999;10(4):35–41.

15. Phare Creative Studio [Internet]. [cited 2019 Dec 12]. Available from: https://pharestudio.org/

16. Havas Champagne [Internet]. [cited 2019 Dec 12]. Available from: https://www.eurocham-cambodia.org/member/276/CHAMPAGNE

17. Chiek C. The Standard of Living of Garment Workers in Cambodia: Its Determinants and Workers' Perception [Internet]. Cambodian Institute for Cooperation and Peace Working Paper No.26. 2008 [cited 2020 Aug 30]. Available from: https://pdfs.semanticscholar.org/f608/a529ae0711be5e7b29970e374ae5b7e1b634.pdf

18. Blomberg M. Mass sackings send chill through Cambodian garment factories [Internet]. Reuters. 2019 [cited 2019 Dec 12]. Available from: https://www.reuters.com/article/us-cambodia-women-garments/mass-sackings-send-chill-through-cambodian-garment-factories-idUSKCN1P41QH

19. ILO. Cambodian Garment and Footwear Sector Bulletin [Internet]. 2018. Available from: https://www.ilo.org/asia/publications/issue-briefs/WCMS_631686/lang--en/index.htm

20. Hootsuite, We Are Social. Digital 2021 Cambodia [Internet]. 2021. Available from: https://datareportal.com/reports/digital-2021-cambodia

21. Smith C, Vannak U, Sokhey L, Ngo T, Gold J, Free C. Mobile Technology for Improved Family Planning (MOTIF): the development of a mobile phone-based (mHealth) intervention to support post-abortion family planning (PAFP) in Cambodia. Reprod Health. 2016;13(1):1–8.

22. Petitet P, Desclaux A. Reproductive health and HIV in Cambodia: from anthropology to public health [Internet]. Groupe de Recherche Cultures, Santé, Sociétés, Paul Cézanne Université, Aix Marseille III (France); 2011. Available from: http://hal.archives-ouvertes.fr/docs/00/58/42/95/PDF/Hancart_Desclaux_2010.pdf

23. Alvergne A, Stevens R, Gurmu E. Side effects and the need for secrecy: characterising discontinuation of modern contraception and its causes in Ethiopia using mixed methods. Contracept Reprod Med. 2017;2(1):24.

24. Chang E, Free C. The factors involved in young women's hormonal contraception decisions. A depth interview study. Contraception. 2005;71(6):426–31.

For peer review only

For peer review only - http://bmjopen.bmj.com/site/about/guidelines.xhtml

25. World Health Organization. Classification of digital health interventions v1.0 [Internet]. 2018 [cited 2020 Aug 30]. Available from: https://www.who.int/reproductivehealth/publications/mhealth/classification-digital-health-interventions/en/
26. Lave J, Wenger E. Situated Learning: Legitimate Peripheral Participation. Cambridge University Press: Cambridge University Press; 1991.
27. Reiss K, Andersen K, Pearson E, Biswas K, Taleb F, Ngo TD, et al. Unintended Consequences of mHealth Interactive Voice Messages Promoting Contraceptive Use After Menstrual Regulation in Bangladesh: Intimate Partner Violence Results From a Randomized Controlled Trial. Glob Heal Sci Pract. 2019;7(3):386–403.
28. Bacchus LJ, Reiss K, Church K, Colombini M, Pearson E, Naved R, et al. Using Digital Technology for Sexual and Reproductive Health : Are Programs Adequately Considering Risk ? 2019;7(4):507–14.
29. Dam R, Teo Y. Personas - A Simple Introduction [Internet]. Interaction Design Foundation. 2019 [cited 2020 Aug 30]. Available from: https://www.interaction-design.org/literature/article/personas-why-and-how-you-should-use-them
30. Marie Stopes International Cambodia. Marie Stopes Cambodia Facebook page [Internet]. 2019 [cited 2019 Dec 12]. Available from: https://www.facebook.com/MarieStopesKh/videos/
31. Marie Stopes International Cambodia. Marie Stopes International Cambodia YouTube channel [Internet]. YouTube. 2020 [cited 2020 Aug 30]. Available from: https://www.youtube.com/channel/UCHIQ85EqjAIyl3tCmuVb0GQ/videos
32. Maries Stopes International. Evidence summary: supporting self-management of MA from pharmacies [Internet]. MSI evidence brief. 2020 [cited 2020 Oct 10]. Available from: https://www.safeaccesshub.org/access/evidence-summary-supporting-self-management-of-ma-from-pharmacies/
33. McCarthy OL, Wazwaz O, Osorio Calderon V, Jado I, Saibov S, Stavridis A, et al. Development of an intervention delivered by mobile phone aimed at decreasing unintended pregnancy among young people in three lower middle income countries. BMC Public Health. 2018;18(1):1–15.

Table 1: Sources of information for the needs assessment

Information domain	Published and grey literature	Observation	Interviews (including discussions)
Factory worker demographics	70% are aged <30 years, half have only primary school education, and most of them have migrated from rural areas away from their family and community support. Higher rate of abortion compared to young women in the national survey, contraception use	Observations of factory workers in and outside of factories consistent with literature	Interviews consistent with literature and observations, although not randomly selected
Factory workers	Grey literature (NGO	Observations in factories	Formal interviews with 33

daily life and living conditions outside the factory	reports and newspaper articles) but very limited in detail. Academic literature focused on specific aspects of health and earnings. Limited qualitative work highlighting living conditions (Chansanphors 2008)	(working space, canteen, infirmary, public spaces) , in shops and markets near factories and in 1 worker's home. This provided a useful perspective in the spaces where workers live, what kind of goods and services they have access to, how far they need to go to access specific services.	female factory workers. "Hanging out" at break times, over meals and at their homes for informal conversations. This allowed us to build rapport, and to contextualize, triangulate and clarify information received from a variety of sources.
Sources of information for family planning and abortion	Literature and grey literature on contraception and abortion needs and services. No research exists on use of web sources for health/sexual health information in Cambodia.	- Direct observation of online activities did not yield any finding regarding searching for family planning information.<br>- Direct search on YouTube for 'family planning' 'abortion' 'medical abortion' 'contraceptive pill' showed several videos on the topics, some instructional some editorial. All videos had comments, many from 2019 (regardless of when the video was first published), which indicate increasing engagement with online sources to look for family planning information.	Interviews with factory workers indicated a strong reliance on family and friends for information related to contraception and abortion. Medical practitioners were also cited as a source of information, but less influential. Interviewees who were asked directly denied looking for family planning information online, but some said they looked for other health information on Facebook or Youtube.
Family planning providers reproductive health practices	Published literature on family planning in Cambodia	Observation in garment factory infirmaries and private providers	Interviews with 22 providers, including factory nurses, pharmacist, private nurses and doctors.

For peer review only

Table 2: Examples of possible interventions

Example(s)	Intervention classification	Main target	Potential benefits	Potential challenges/disadvantages
Edutainment video about contraception	Targeted client communication (transmit targeted health information to client based on health status or demographics)<br>Goal is to increase awareness, not to instruct on specific	Clients (potential or existing users of contraception, factory workers)	Potential for increased reach<br>Potential to influence attitudes and behaviour about contraception use	Does not provide detailed information about pros and cons of contraceptive method

For peer review only - http://bmjopen.bmj.com/site/about/guidelines.xhtml

	details of contraceptive methods			
Provider-to-client communication	Targeted alerts/reminders on mobile phones	Existing clients	Increase in follow-up visits and continuity of care<br>Increase in adherence to instructions	Privacy (if phones are shared or anyway accessible to other people)<br>Frequent changes of numbers and loss of phones make it difficult to have unique phone ID for unique clients
Provider to provider communication	Increase sharing of best practices in informal private online groups (e.g. Whatsapp and Facebook group chats)	Providers, especially private providers who do not have regular opportunities for professional updates and training	- Provide informal opportunities for sharing best practices and asking for advice<br>- Leverage social and informal ties to create strong 'communities of practice' (Lave & Wenger, 1991)	- Informal groups can be difficult to sustain without participants' buy-in; need a few motivated individuals.<br>- Peer-to-peer information exchanges are not necessarily medically sound, so they could help spread misinformation
Instructional video about contraception methods	On-demand information services to clients	- Existing clients<br>- Potential users already considering a specific method<br>- Providers who need reminding/training about how specific methods work	- Video more attractive format compared with written text, and already a common source of information among targeted audiences.<br>- Can reach clients who are not comfortable or able to go to pharmacies/clinics<br>- comments to the videos can offer insights into frequent questions, and potentially serve as a source of referral for clinics.	Unclear how likely videos are in influencing attitudes and behaviour<br>Rely on being found in the midst of other commercial videos fighting for attention<br>Rely on users having the connectivity to watch the video<br>Requires resources to produce, post, and keep updated<br>Requires resources to potentially monitor and answer comments and questions
Video about abortion	Education about abortion	- - Clinic/pharmacy clients<br>- Women at risk of or with unintended pregnancy	- Can reach audiences that are uncomfortable with text and/or with visiting clinics to ask for information<br>- Can offer a medically sound perspective, among propaganda and medically dubious videos currently available online.	- Difficult topic to engage with through an accurate, but accessible and engaging video<br>- Requires significant effort in managing the online presence of such videos (moderation, reliability, findability among competing

For peer review only - http://bmjopen.bmj.com/site/about/guidelines.xhtml

				anti-abortion videos, etc)
Instructional video about medical abortion	Education about MA	- Women with unintended pregnancy	- More accessible alternative to written text for those with low-literacy levels.<br>- Can be easier to access in private than written leaflets.	- Needs to be found online, against existing competing videos that might be less accurate but are ranked higher in search results<br>- Privacy issues, as it remains in search history

Table 3: Key metrics of the three videos one month after release

	**Mother**	**Love**	**Baby (shortened)**
Video release	13 Aug 2019	Sept 13, 2019	11 Oct 2019
Boost budget	$1,016	$1,016	$1,166
Video plays	2,268,736	2,834,282	2,704,121
Engagement and rate (e.g. likes, shares, comments)	679,591	1,265,398	873,388
Engagement rate	20%	38%	24%
Reach (nationwide)	1,406,274	1,307,140	1,305,075
Cost per reach (CPR) (nationwide)	$0.30	$0.32	$0.32
Click to Action (CTA) (*send message to Marie Stopes)	3,997	23,700	4,728
Cost per click (CPC)	$0.03	$0.01	$0.03

Definitions: Reach is the number of people who saw a post at least once. Engagement is the number of people interacting with the content. Example is like, share, comment, reactions. Click to Action measures the number of people who click the "Send message" button that will lead to MSIC Facebook messenger.

Figure legends

Figure 1: London and Phnom Penh Fieldwork Timeline

Figure 2: Logic model of the problem

Figure 3: Contact centre total calls and Facebook messages (inbound and outbound)

For peer review only

For peer review only - http://bmjopen.bmj.com/site/about/guidelines.xhtml

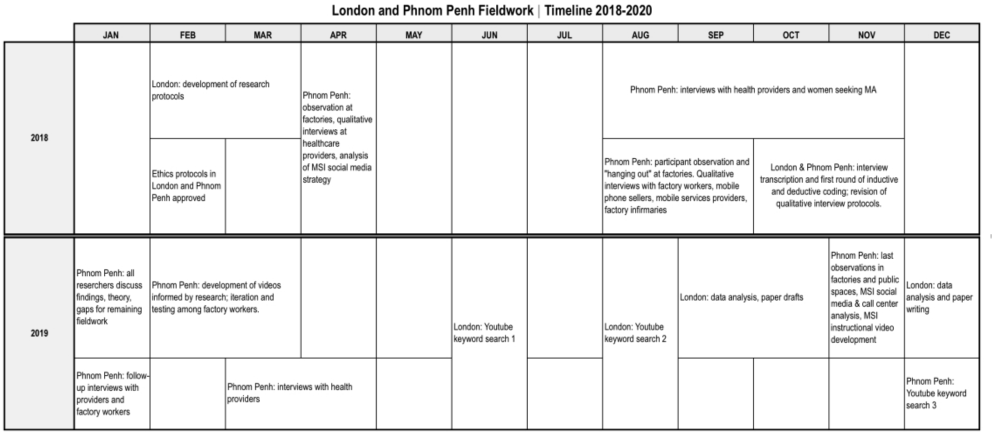

London and Phnom Penh fieldwork timeline

210x93mm (300 x 300 DPI)

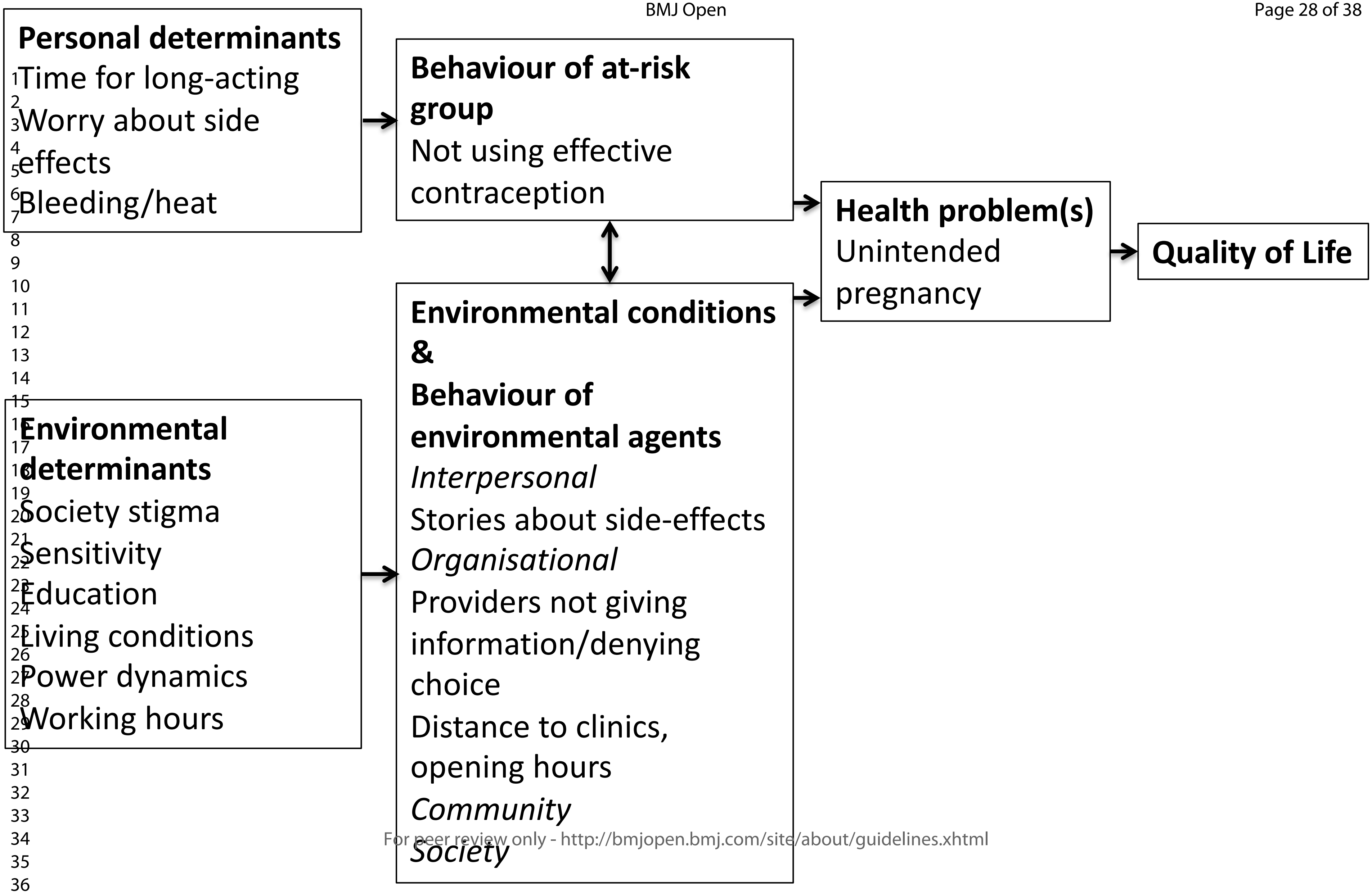

For peer review only - http://bmjopen.bmj.com/site/about/guidelines.xhtml

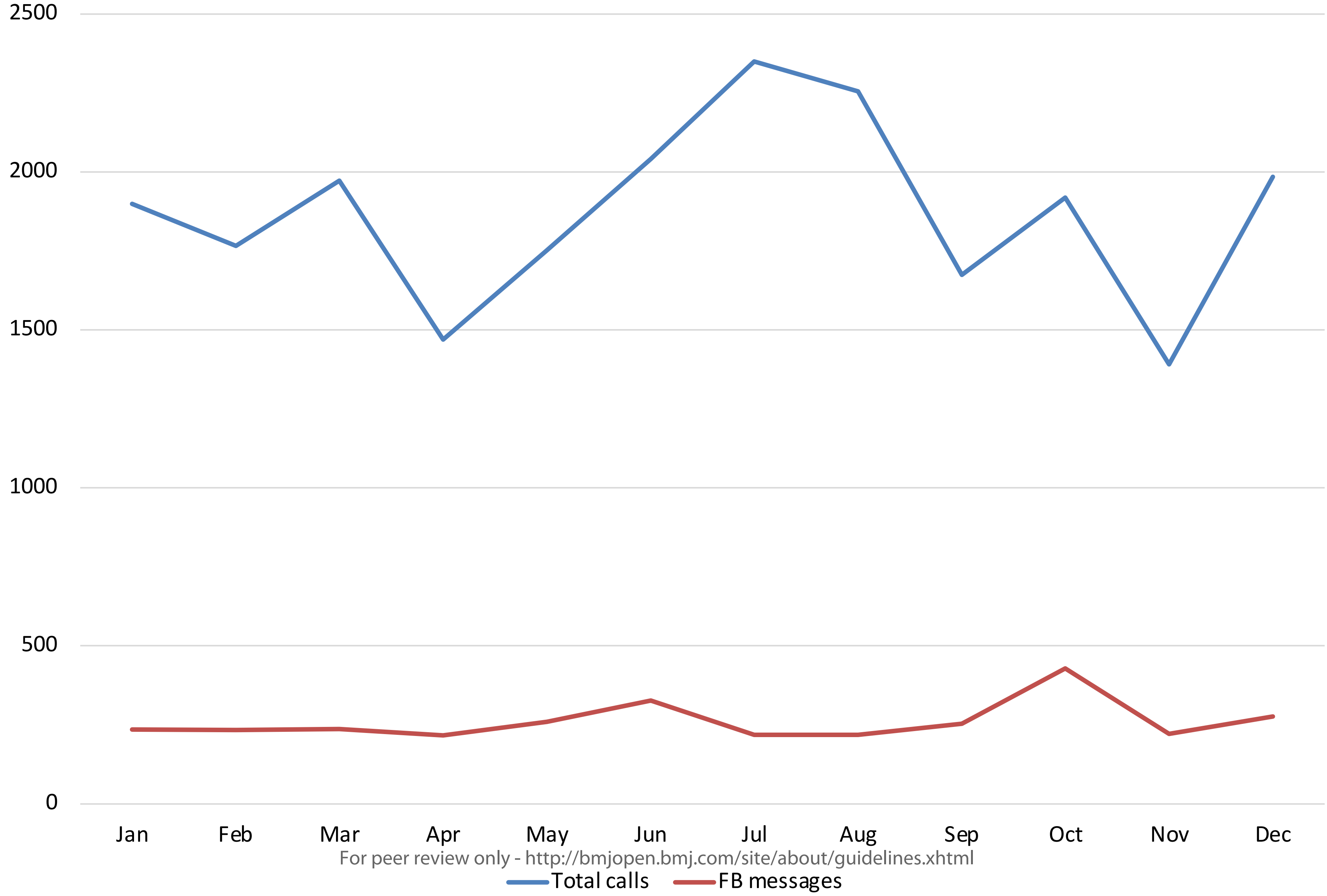
2500
2000
1500
1000
500
0
Jan
Feb
Mar
Apr
May
Jun
Jul
Aug
Sep
Oct
Nov
Dec
Total calls
FB messages

Appendix A: Interview guide for women seeking MA

Background information

What is your age?
Where are you from?
How long have you been in Phnom Penh?
Where do you work and what is your role? (Don't need specifics of factory)
How is your work?

Relationships and living situation

Are you married?
If no: do you have a relationship?
Do you have children?
How many?
How old are they?
Where do they live?
What sort of accommodation do you live in?
Who do you live with?

MA decision-making, expectations and information seeking

Have you had an abortion before?
When did you realise you were pregnant this time?
How did you realise you were pregnant?
What happened after that?<br>Feelings<br>Further confirmation sought<br>Who discussed it with<br>Why decided to have an abortion this time<br>Do you think this is a common reason for women to have abortion here?
How did you decide what to do?<br>Did you talk to your family or friends?

For peer review only

How did you make the decision?
Why did you choose this provider?
How did you learn about MA?<br>Do you have any friends or family who have had MA?<br>What was their experience?
Why did you choose MA instead of another way?

If have not taken MA yet:

Did you come with anyone today?
When do you think you will take the MA?
What do you expect to happen when you take the MA?
Have you been told will happen?<br>Who has told you this?<br>What has the provider said?
How will you manage it?
What will you do if you need more advice?
Will you need to take any time off work?
What are your feelings about taking it?

If have taken MA already:

Did you come with anyone to get the MA?
When did you take the MA?
What happened next?<br>If any decisions made or further care sought: ask about these and why
How did you manage the bleeding?<br>Did you use any products?<br>How much did these cost?
Did you take any time off work?
Was the MA process what you expected?<br>What did the provider tell you?

For peer review only

For peer review only - http://bmjopen.bmj.com/site/about/guidelines.xhtml

Did anyone else tell you what to expect?
What were your feelings about taking MA?
Did anyone help you at home?

Attitudes and barriers around MA

Was it easy or hard to come to buy MA?<br>Why was it easy/hard?<br>What would have made it better for you?
Who would you tell and who would you not tell?<br>Would you tell your friends?
Do people here think abortion is a bad thing?<br>Who says this?

If have not taken MA yet:

Do you know how much the cost is?<br>Will anyone help you pay?
Did you have to pay for transport here?

If have taken MA already:

What were the biggest challenges to taking MA?
How much did the MA (/MVA) cost?<br>Did anyone help you pay?<br>Did you have to pay for anything else? Transport?

Contraception

Have you used contraception before?
If yes: which methods?<br>How long for?<br>What was your experience of it?<br>Why did you stop using it?
If no: why do you not use contraception?

For peer review only

Do you plan to use any contraception after the abortion?
If yes: do you know which method you will have?<br>Do you know when you will get it?<br>Do you know where?<br>*If reported side effects:* do you know what you will do if you get the side effects?
If no: why not?<br>How will you prevent pregnancy?<br>Do you know which methods are available?
How do you make decisions about FP?<br>Where would you get information about FP?<br>Have you ever seen any videos or information online about FP?<br>Do you and your friends ever talk about contraception?<br>What contraception do your friends use?<br>What do they say about it?

General life questions

What are your plans for the future with your family and your work?
Do you have a mobile phone?<br>What type is it?<br>What do you use it for?
How do you enjoy yourself on your day off?

Is there anything you want to ask us?

For peer review only - http://bmjopen.bmj.com/site/about/guidelines.xhtml

Appendix B: Interview guide for private providers

MA provision

How long they have been providing MA
Available types of drug for MA
Approximate number of clients per week
Information given to clients

Training

What formal training they have been given
How they keep up to date
What support they would like

Post-abortion care and support

Post-abortion issues encountered
Post-abortion care provided
Suggestions for interventions

For peer review only

For peer review only - http://bmjopen.bmj.com/site/about/guidelines.xhtml

Supplementary file 2: video scripts

LOVE VIDEO: https://youtu.be/nVOSnsLvrBY

Character	Script
BOPHA:	She is not married yet! Hmm... I would be afraid of getting pregnant!
SAMBO:	If I was her, I will use Condom in this situation.
MARDI:	But if you forgot to take the condoms. For me I would use injection every 3 months, so I do not worry about pregnancy anymore! Nobody knows I'm using it. It's easy to enjoy the romance.
LINDA:	I feel more confident by using the pill. My mother used it before she had me. It did not affect her health at all...
BOPHA:	... at least she had a very beautiful daughter!
HEANG:	I chose implant! Here it is! Implants are highly effective up to 3-5 years and discreet. I was not afraid to choose it as my older sister used it before. I will ask a provider to remove it only when I want to have a baby.
BOPHA:	Sorry girls, I have never used anything you talk about. I worry that in a spontaneous situation, I don't have any way to avoid unintended prenancy. Using contraception for protection is better than abortion.
Kunthea:	Hope is not a safe plan.This is my first child. I don't want to have another one right now. We need sometime to save money. You know, why I use IUD. IUD provides protection for up to 10 years long.It is small flexible plastic devide inserted into uterus. and it does not release Hormom like other method and i do not worry about pregnant when my husband back home i still have romance love as a couple on the rice field.
LADY IN RICEFIELD:	What method you choose is your choice. Keep calm and chose the contraceptive methods that fit the most with your life. Do not worry about getting pregnant!
VOICE OVER:	For more information about finding the right method that fits with for your health, or if contraception goes wrong and you need emergency contraception, call Marie Stopes to speak to an adviser<br>012 999 002/098 999 102 . Or find us on FB at https://www.facebook.com/MarieStopesKh

BABY DANCING VIDEO: https://youtu.be/RhmXnc-lvNs

Character	Script
Doctor:	If you have any issue, come and talk to us in order to find a solution
Lady:	I feel hot and cold. Do not want to use pill. Even when I use it, don't use it regularly.
Doctor:	If you don't feel good, you should prevent in advance. Don't be careless to have unintended pregnancy. Prevention is better.
Lady:	It seems too early. It is weird for me without husband but using contraception
Doctor:	You have the choice, between injection, implant, IUD and condom. it's simple and easy.
Lady:	I am satisfied taking the pill but I have to know that I have to take it everyday. Thank you doctor for accurate information! Now I have no doubt, less anxiety to use contraception and there are many family planning methods for everybody. You have to think and choose correctly. Don't let yourself have sadness or bad problem because do not use any prevention.
Doctor + lady:	Nothing is easier than prevention. It make you have a happy family and a better future.
Voice over:	For more information about finding the right method that fits with for your health, or if contraception goes wrong and you need emergency contraception, call Marie Stopes to speak to an adviser 012 999 002/098 999102 . Or find us on FB at https://www.facebook.com/MarieStopesKh

MOTHER VIDEO: https://youtu.be/1GzGDI61Ssc

Character	Script
YOUNG BROTHER:	What's wrong mum?
MUM:	I just feel dizziness, nausea and hot

For peer review only - http://bmjopen.bmj.com/site/about/guidelines.xhtml

YOUNG BROTHER:	Why? Are you sick Mum?
MUM:	I'm fine, don't worry, I am not sick at all, ...I just take medicine to prevent having another child
SISTER:	Mum wants to say that she took contraceptive' pills
SISTER:	You must take 1 pill every day regularly for it towork. It contains 2 hormones, similar to those that are already found in every woman's body.
YOUNG BROTHER:	Ah...I see. Do you have any side effects Mum?
MUM:	Yes I do son.
SISTER:	Don't worry Mum. The side-effects often go away after 2-3 months. Not everybody gets side effects like you. Do you know, it makes woman have regular periods, and could make you have nice skin and clear acne.
YOUNG BROTHER:	Are there any other methods that more effective and safe?
SISTER:	Well I see 3 more options. Isn't it Mum?
SISTER:	Injections are effective for 3 months. Nobody knows you are using it. You might have some irregular bleeding at first then you might have lighter bleeding. This happens because the uterus lining is not building up. It is normal to disapear over time. It does not affect your health.
SISTER:	If you want a long term family planning Implant is a good choice. It is effective for 3-5 years. It is a small soft plastic pipe. It takes the provider only 5 minutes to insert Implant to put it under arm skin or remove it. And when you want to have another child you just go to see provider to remove it. When it is take out you can have the baby again as normal.
SISTER:	The Intra Uterine Device or IUD is effective for 10 years. It is small and does not release hormones. It stops sperm from meeting the eggs to fertilise. No surgery is required to insert and remove IUD. It makes woman have regular periods.
MUM:	Thank you kids for all your advises!
BABY:	Don't worry Mum! Be confident with your choice for our future. We are here supporting you. Now you know there are other contraception methods for you to choose.
VOICE OVER:	For more information about finding the right method that fits with for your health, or if contraception goes wrong and you need emergency contraception, call Marie Stopes to speak to an adviser<br>012 999 002/098 999102 . Or find us on FB athttps://www.facebook.com/MarieStopesKh

For peer review only

Standards for Reporting Qualitative Research (SRQR)*

http://www.equator-network.org/reporting-guidelines/srqr/

Title and abstract

	Page/line no(s).
**Title** - Concise description of the nature and topic of the study Identifying the study as qualitative or indicating the approach (e.g., ethnography, grounded theory) or data collection methods (e.g., interview, focus group) is recommended	Page 4/Line 88
**Abstract** - Summary of key elements of the study using the abstract format of the intended publication; typically includes background, purpose, methods, results, and conclusions	Page 2/Lines 35-62

Introduction

**Problem formulation** - Description and significance of the problem/phenomenon studied; review of relevant theory and empirical work; problem statement	Page 4-5/Lines91-118
**Purpose or research question** - Purpose of the study and specific objectives or questions	Page 3-4/Line 115-118

Methods

**Qualitative approach and research paradigm** - Qualitative approach (e.g., ethnography, grounded theory, case study, phenomenology, narrative research) and guiding theory if appropriate; identifying the research paradigm (e.g., postpositivist, constructivist/ interpretivist) is also recommended; rationale**	Pages 4-6/Lines 124-148 in methods: ethnography and interviews rationale, constructivist data analysis
**Researcher characteristics and reflexivity** - Researchers' characteristics that may influence the research, including personal attributes, qualifications/experience, relationship with participants, assumptions, and/or presuppositions; potential or actual interaction between researchers' characteristics and the research questions, approach, methods, results, and/or transferability	Page 5/Lines 126-132, methods<br><br>Page 17/Line 565-589 discussion
**Context** - Setting/site and salient contextual factors; rationale**	Pages 4-5/Lines 91-114 background. Pages 7-8/Lines 227-267, Research context
**Sampling strategy** - How and why research participants, documents, or events were selected; criteria for deciding when no further sampling was necessary (e.g., sampling saturation); rationale**	Page 6/Lines 154-182, methods

For peer review only - http://bmjopen.bmj.com/site/about/guidelines.xhtml

**Ethical issues pertaining to human subjects** - Documentation of approval by an appropriate ethics review board and participant consent, or explanation for lack thereof; other confidentiality and data security issues	Page 7/Lines 212-218, Ethics
**Data collection methods** - Types of data collected; details of data collection procedures including (as appropriate) start and stop dates of data collection and analysis, iterative process, triangulation of sources/methods, and modification of procedures in response to evolving study findings; rationale**	Pages 5-7/Lines 154-182, methods,
**Data collection instruments and technologies** - Description of instruments (e.g., interview guides, questionnaires) and devices (e.g., audio recorders) used for data collection; if/how the instrument(s) changed over the course of the study	Pages 5-7/Lines 142-147, 174-176, methods,
**Units of study** - Number and relevant characteristics of participants, documents, or events included in the study; level of participation (could be reported in results)	Pages 5-7/Lines 156-158, 167-172, 177-180, methods,
**Data processing** - Methods for processing data prior to and during analysis, including transcription, data entry, data management and security, verification of data integrity, data coding, and anonymization/de-identification of excerpts	Page 5/Lines 142-143, methods,
**Data analysis** - Process by which inferences, themes, etc., were identified and developed, including the researchers involved in data analysis; usually references a specific paradigm or approach; rationale**	Pages 5-7/Lines 143-147, 182, methods
**Techniques to enhance trustworthiness** - Techniques to enhance trustworthiness and credibility of data analysis (e.g., member checking, audit trail, triangulation); rationale**	Page 5/Lines 142-147 - methods - coding

Results/findings

**Synthesis and interpretation** - Main findings (e.g., interpretations, inferences, and themes); might include development of a theory or model, or integration with prior research or theory	Pages 11-12/Lines 354-376
**Links to empirical data** - Evidence (e.g., quotes, field notes, text excerpts, photographs) to substantiate analytic findings	Page 6/Lines 182

Discussion

**Integration with prior work, implications, transferability, and contribution(s) to the field -** Short summary of main findings; explanation of how findings and conclusions connect to, support, elaborate on, or challenge conclusions of earlier scholarship; discussion of scope of application/generalizability; identification of unique contribution(s) to scholarship in a discipline or field	Pages 15-17/Lines 482-555
**Limitations** - Trustworthiness and limitations of findings	Page 17/Lines 558-576

Other

**Conflicts of interest** - Potential sources of influence or perceived influence on study conduct and conclusions; how these were managed	Page 19/Line 635

For peer review only

**Funding** - Sources of funding and other support; role of funders in data collection, interpretation, and reporting	Page 19/ Lines 637-640

*The authors created the SRQR by searching the literature to identify guidelines, reporting standards, and critical appraisal criteria for qualitative research; reviewing the reference lists of retrieved sources; and contacting experts to gain feedback. The SRQR aims to improve the transparency of all aspects of qualitative research by providing clear standards for reporting qualitative research.

**The rationale should briefly discuss the justification for choosing that theory, approach, method, or technique rather than other options available, the assumptions and limitations implicit in those choices, and how those choices influence study conclusions and transferability. As appropriate, the rationale for several items might be discussed together.

Reference:
O'Brien BC, Harris IB, Beckman TJ, Reed DA, Cook DA. **Standards for reporting qualitative research: a synthesis of recommendations.** *Academic Medicine*, Vol. 89, No. 9 / Sept 2014 DOI: 10.1097/ACM.0000000000000388

For peer review only
